# Modulation of TCR signalling components occurs prior to positive selection and lineage commitment in iNKT cells

**DOI:** 10.1038/s41598-021-02885-w

**Published:** 2021-12-08

**Authors:** Xuyen T. Dinh, Dragana Stanley, Letitia D. Smith, Morgane Moreau, Stuart P. Berzins, Adrian Gemiarto, Alan G. Baxter, Margaret A. Jordan

**Affiliations:** 1 Molecular & Cell Biology, College of Public Health, Medical & Veterinary Sciences, The Science Place, Building 142, James Cook University, Townsville, QLD 4811 Australia; 2 Hai Duong Medical Technical University, Hai Duong, Viet Nam; 3 School of Medical and Applied Sciences, Central Queensland University, Rockhampton, QLD 4702 Australia; 4 School of Science, Psychology and Sport, Federation University Australia, Ballarat, VIC 3350 Australia; 5 Peter Doherty Institute for Immunity and Infection, University of Melbourne, Parkville, VIC 3050 Australia

**Keywords:** Cell biology, Genetics, Immunology, Molecular biology, Systems biology, Diseases

## Abstract

iNKT cells play a critical role in controlling the strength and character of adaptive and innate immune responses. Their unique functional characteristics are induced by a transcriptional program initiated by positive selection mediated by CD1d expressed by CD4^+^CD8^+^ (double positive, DP) thymocytes. Here, using a novel Vα14 TCR transgenic strain bearing greatly expanded numbers of CD24^hi^CD44^lo^NKT cells, we examined transcriptional events in four immature thymic iNKT cell subsets. A transcriptional regulatory network approach identified transcriptional changes in proximal components of the TCR signalling cascade in DP NKT cells. Subsequently, positive and negative selection, and lineage commitment, occurred at the transition from DP NKT to CD4 NKT. Thus, this study introduces previously unrecognised steps in early NKT cell development, and separates the events associated with modulation of the T cell signalling cascade prior to changes associated with positive selection and lineage commitment.

## Introduction

The innate-like population of T cells, iNKT, rapidly respond to both cytokine and TCR stimulation by the production of pro-inflammatory and immunoregulatory cytokines. Their scarcity in many species belies their significance, as when dysfunctional or deficient, they can influence autoimmune, allergic and malignancy outcomes [reviewed in^1^]. In the periphery of mice, they express a ‘memory’ or ‘activated’ like surface phenotype (CD62L^−^CD69^+^CD44^hi^) and the great majority are either CD4^+^CD8^−^ (single positive; SP) or CD4^−^CD8^−^ (double negative; DN)^2,3^. They are functionally heterogeneous, and so far, iNKT1, iNKT2, iNKT17, iNKTreg, iNKT_FH_, and iNKT10 subsets have been described, classified according to transcription factor (TF) and cytokine expression profile^4–7^, with iNKT1, iNKT2 and iNKT17, mirroring the respective Th cell subsets in cytokine production, while others are specialised in T follicular helper-like function (iNKT_FH_), have IL-10-dependent regulatory functions (iNKT10) or regulatory functions resembling Tregs (iNKTreg). In vivo iNKT responses are likely dependent on the subsets and locations of iNKT cells activated. The lineage commitment and subsequent development of these cells have been subjects of considerable interest. iNKT cells are not found in the peripheral tissues of mice until 1–2 weeks after birth and their development is thymus dependent. Consistent with this, iNKT cells develop in fetal thymic organ culture^8,9^, and neonatal thymectomy on the third day of life selectively depletes them^10^.

While conventional αβT cells express a diverse repertoire of TCR sequences that are generated by random rearrangement^11–13^ and are positively selected by thymic epithelium expressing MHC Class I and Class II products^14^, Type 1 NKT cells express a highly restricted (semi-invariant) TCR (Vα14 Jα18 in mice, and the homologous Vα24 Jα18 chain in humans, paired with a restricted range of β chains) and the vast majority of them are positively selected by ligating β-2 microglobulin/CD1d^8,15–19^ and SLAM family members^20–24^ expressed on DP cortical thymocytes^25–27^. A small fraction of the DNNKT cells are believed to branch off earlier and be derived from late stage TCRβ^+^CD4^−^CD8^−^ double negative thymocytes^28^.

In iNKT cell development, much attention has been paid to the acquisition of the NK1.1 marker, primarily because prior to the development of CD1d tetramers (CD1d-tet)^2,3^, expression of NK1.1 on CD3^+^ cells was used as a surrogate marker of iNKT cells. Although mouse thymi contain CD44^hi^NK1.1^+^CD1d-tet^+^ NKT cells, many of these appear to be non-dividing and long-lived^9,29,30^. In contrast, recent thymic emigrant iNKT cells are enriched for CD44^hi^NK1.1^−^ and acquire NK1.1 expression after thymic egress^30,31^.

In an attempt to identify the earliest CD1d-tet^+^ thymic iNKT cells, Gapin et al.^29^ stained C57BL/6 mouse thymi with CD1d-tet-phycoerythrin (PE), and enriched positive cells with anti-PE magnetic beads. Of the NKT cells obtained from thymi of nine-day old mice, 25% were “DP^dull^”, a population whose frequency declined with age, was not seen in the periphery at any age, and was absent in CD1d^−/−^ mice. This population probably represented early post-selection iNKT cells^29^, because, in conventional T cell development, TCR ligation of DP^hi^ thymocytes triggers CD4 or CD8 down-regulation^32,33^. In contrast, equivalent proportions of DP^hi^ thymocytes were identified in wild type (WT) and *Cd1d*^−*/*−^ mice, by quantitative polymerase chain reaction (PCR) analysis of Vα14-Jα18 encoded transcripts of sorted cells, consistent with the DP^hi^ phenotype marking pre-selection iNKT cells^29^. Further evidence of the DP^hi^ to DP^dull^ transition representing positive selection was obtained by CDR3 spectratyping; in WT mice, only a single peak could be detected in the DP^dull^ population, which corresponded to the canonical Vα14-Jα18 rearrangement. This contrasted with the multiple rearrangements detected in DP^hi^ thymocytes^29^.

Benlagha^34^ applied a similar tetramer-based enrichment strategy to identify DP CD24^hi^ NK1.1^−^ NKT cell precursors in the thymi of new-born mice. These cells were non-dividing and already exhibited the same Vβ8 TCR bias characteristic of mature NKT cells. They therefore are likely to be post-selection, making them the earliest population of post selection iNKT cells identified to date. In contrast, the subsequent population of CD24^lo^NK1.1^−^ CD1d-tet^+^ iNKT cells contained two subsets (CD44^lo^ and CD44^hi^), both of which were rapidly proliferating^30^. On the basis of the changing proportions with aging from two- to- six weeks, Benlagha et al.^30^ proposed that the CD44^lo^ population precedes the CD44^hi^ population in maturation. The postulated developmental pathway of iNKT cells in mice is therefore:

DP^hi^CD24^hi^CD44^lo^NK1.1^−^ → selection → DP^lo^CD24^hi^CD44^lo^NK1.1^−^ → CD4^+^CD24^hi^CD44^lo^NK1.1^−^ (these earliest detectable populations of CD24^hi^CD44^lo^NK1.1^−^ cells are referred to as Stage 0) → DN or CD4^+^ CD24^lo^CD44^lo^NK1.1^−^ (Stage 1) → DN or CD4^+^CD24^lo^CD44^hi^NK1.1^−^ (Stage 2) → DN or CD4^+^ CD24^lo^CD44^hi^NK1.1^+^ (Stage 3).

Given the rarity of DP^lo^ CD24^hi^ cells, Benlagha et al.^34^ suggested it is not surprising that, for technical reasons, the presence of DP^hi^ precursors could not be detected, and that their rarity supports the hypothesis that Vα14-Jα18 rearrangements occur stochastically and at low frequency.

An understanding of the mechanisms of iNKT cell subset differentiation is likely a prerequisite of the manipulation of the iNKT cell response for therapeutic purposes. To date, attempts to characterise these have mainly queried common factors or reaffirmed the roles of specific factors that were referred from conventional T cell development, or identified in relatively mature iNKT cells. This is primarily due to the scarcity of very immature iNKT cells, making the analysis of the early stages in the transcriptional programs controlling iNKT cell development very challenging. We have attempted to address this problem by the production of a transgenic mouse model with increased numbers of immature iNKT cells generated on NOD background. While NOD mice are characterised by reduced numbers, and relative immaturity, of iNKT cells^35,36^, and these features contribute to their susceptibility to type 1 diabetes^37–39^, activation of Vα14^+^ NKT cells by α-Galactosylceramide (αGalCer)^40^ or increasing iNKT cell numbers by the transgenic introduction of the rearranged *V*α*14-Ja18* TCR gene segment increased iNKT cell numbers and prevented disease^41^.

In the work described in this manuscript, Vα14Jα18 transgenic NOD mice were created by directly injecting NOD/Lt embryonic pronuclei with a transgenic construct containing an NKT-associated Vα14-Jα18 TCRα chain cDNA under the regulatory control of a minimal CD4 promoter, enhancer and intronic silencer^22^. Throughout the remainder of this paper, the strain designation “NOD” refers to NOD.*Nkrp1b*^*b*^ mice and “NOD.*Va14*^*tg*^” refers to NOD.*Nkrp1b*^*b*^.*Va14*^*tg*^ mice. NOD.*Va14*^*tg*^ mice were found to have greatly increased numbers of iNKT cells with the characteristics of pre-selection and Stage 0 iNKT cells. This model therefore provided an opportunity to dissect the transcriptional programs involved in iNKT cell positive selection and lineage commitment, prior to Stage 1, in this stain, and has, in conjunction with other approaches, helped to shed light on the early events in iNKT cell development.

## Materials and methods

### Mice

NOD.*Nkrp1b*^*b*^, C57BL/6 J, congenic, transgenic and mutant mice were maintained at the Immunogenetics Research Facility at the James Cook University under SPF conditions. Mice of both sexes were analysed at six-to-eight weeks of age unless otherwise noted. Experimental mice were age and sex–matched with controls and experiments were repeated 2–3 times.

The NOD.*Nkrp1b*^*b*^ strain carries B6-derived alleles at the Natural Killer Complex on chromosome 6 (from *D6mit105* to *D6mit135*), permitting the use of the NK1.1 marker of NK and NKT cell maturation^36,42^.

CD1d-deficient NOD mouse lines were created by crossing NOD.*Nkrp1*^*b*^ or NOD Vα14-Jα18 transgenic mice to the NOD.129S6-*Cd1d1*^*tm1Luc*^ line^43^, which was a kind gift of Prof Luc Van Kaer, Vanderbilt University School of Medicine. F1 stock were then intercrossed, the progeny genotyped and homozygous founders selected for the propagation of the new lines.

**Figure 1:**
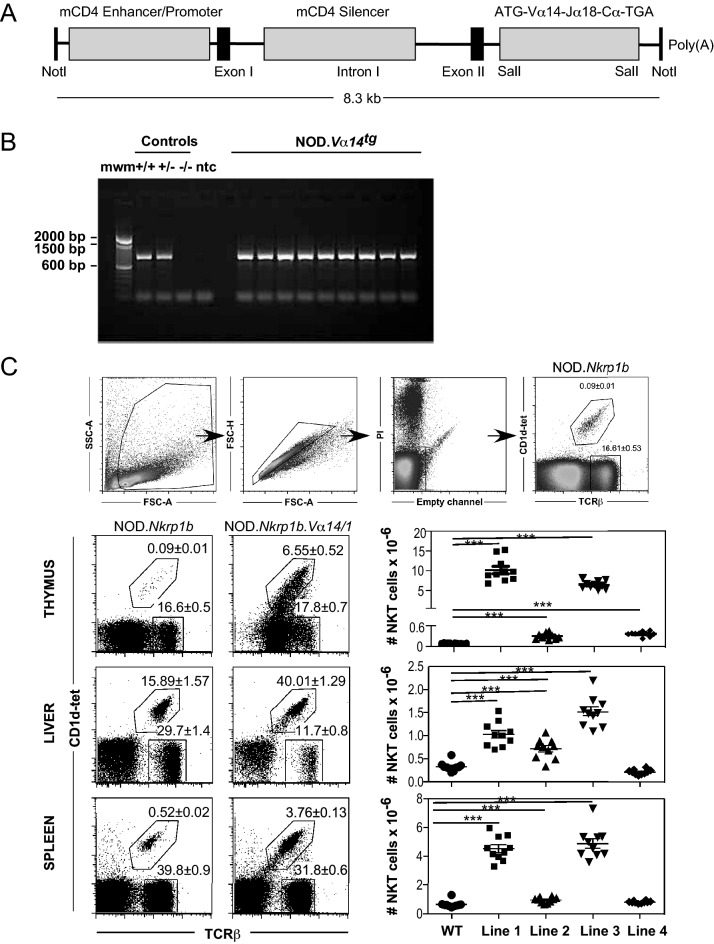
Production and characterisation of NOD.*Va14*^*tg*^ mice. (**A**) A diagram of the transgenic construct, which consists of a CD4 minigene, including a minimal CD4 promoter/enhancer and the CD4 intronic silencer 5’ of Vα14-Jα18 TCRα chain cDNA and a poly-A tail^17^. (**B**) Pups were genotyped by using construct-specific primers. mwm = molecular weight marker; ntc = no template control. Results are shown for nine individual transgenic mice from NOD.*Va14*^*tg*^ Line 1. (**C**) Thymic, hepatic and splenic NKT cell phenotyping of NOD.*Va14*^*tg*^ Lines 1–4 transgenic mice compared to WT NOD mice (n = 10: Data means (± SEM) from 5 males and 5 females for each group. Data are combined from two independent experiments and NKT gating is indicted at the top for WT NOD mice).

NOD Vα14-Jα18 transgenic mice were generated using the previously published construct kindly provided by Prof Albert Bendelac, University of Chicago^22^. Briefly, the prerearranged Vα14-Jα18 TCRα chain complementary DNA (cDNA) of the DN32.D3 hybridoma^44^ was inserted into the SalI site of a plasmid containing the minimal CD4 promoter and enhancer and the intronic silencer^45^ (Fig. 1A). The linearized (via NotI) construct was purified by agarose gel electrophoresis, agarase treated, re-purified and injected directly into NOD/Lt embryonic pronuclei at the Walter and Eliza Hall Institute microinjection unit (Melbourne, Australia). The manipulated embryos were placed in the reproductive tracts of pseudopregnant NOD/Lt recipient female mice. Transgenic mice were screened with polymerase chain reaction (PCR) (forward primer: 5′-TGTAGGCTCAGATTCCCAACC-3′; reverse primer: 5′-GAGGATGGAGCTTGGGAGTCAGG-3′) and crossed onto the NOD.*Nkrp1b*^*b*^ line to permit the use of the NK1.1 developmental marker.

These studies have been reviewed and approved by the James Cook University Animal Ethics Committee. The study was carried out in compliance with the ARRIVE guidelines (https://arriveguidelines.org) and all methods were performed in accordance with the relevant guidelines and regulations.

### Cell suspensions

As previously described^24^, thymocyte and splenocyte cell suspensions were prepared by gently grinding the organ between frosted microscope slides in FACS buffer (PBS containing 2 mM EDTA (Amresco, Solon, Ohio, USA), 10% (v/v) Bovine Serum (Invitrogen, Melbourne, Australia) and 0.02% (w/v) Sodium Azide). Spleen cell suspensions were treated with Red Blood Cell Lysing Buffer (Sigma Aldrich, Castle Hill, NSW, Australia). Viability was generally > 95%.

Livers were harvested after being perfused with 5–10 ml PBS then minced through a 180 um wire stainless steel mesh. The dissociated material was centrifuged at 500 X g for 5 min at 4 °C. Parenchymal cells were removed over a 33.75% Percoll (GE Healthcare, NSW, Australia) gradient. Liver lymphocytes were collected after lysing red blood cells.

### Flow cytometry

For flow cytometric analyses, cells were labelled with anti-TCRβ-APC or PerCP Cy5.5 (clone H57-597), anti-CD4-FITC or V500 (clone RM4-5), anti-NK1.1-PE-Cy7 or PerCP Cy5.5 (clone PK136), anti-CD8-FITC or APC-Cy7 (clone 53–6.7), anti-CD44-V450 or FITC (clone IM7) and anti-CD24-FITC (clone M1/69) (from BD Pharmigen, San Diego, CA, USA). Mouse CD1d tetramer, conjugated to PE and loaded with α-GalCer, was produced by Mr Marcin Ciula (University of Melbourne) using recombinant baculovirus encoding polyhistidine-tagged mouse CD1d and mouse β2 microglobulin, originally kindly provided by Prof M. Kronenberg’s laboratory (La Jolla Institute for Allergy and Immunology, San Diego, CA)^3^.

For surface staining, antibodies were diluted in FACS buffer. As described in Jordan et al.^24^, cells were pre-incubated with unconjugated anti-CD16/32 (clone 93, eBiosciences, San Diego, CA, USA) before addition of surface staining antibody cocktails. Data were acquired on a BD LSRII Fortessa (BD Bioscience, San Jose, California, USA) flow cytometer and analysed using FlowJo software (Tree Star, Inc., Ashland, OR, USA).

### Cell sorting

Single cell suspensions were stained on ice for 25 min with specific antibodies (anti-TCRβ (H57-597), CD1d-tetramer, anti-CD4 (RM4-5), anti-CD8 (53–6.7), anti-CD24 (M1/69) and anti-NK1.1 (PK 136). Cells were sorted on a BD FACSAria II. Cell doublets were excluded by 3 comparisons (forward scatter-area to forward scatter-height, forward scatter-height to forward scatter-width and side scatter-height to side scatter-width). Dead cells were excluded from analysis by propidium iodide staining. All samples were analysed after sorting to confirm viability and cell purities were over 97%.

### In vivo treatment with α-GalCer

Alpha-GalCer was prepared for injection by sonication in PBS with 0.5% Tween-20 for 2 h at 37 °C. Six-week old mice were injected either intravenously (4 μg in 200 μl) or intrathymically (2 μg in 10 μl) with α-GalCer or control vehicle. Mice were either bled four hours later for cytokine analyses or culled 40 h after injection for flow cytometric analyses.

### Microarray analyses

Sorted immature thymic T and iNKT cell subsets (7 samples/group) were individually homogenized in the RLT buffer of an RNeasy kit (QIAGEN, Venlo, Limburg, Netherlands); homogenates were passed through QIAshedder columns (QIAGEN, Limburg, Netherlands) and extracted (RNeasy; QIAGEN, Limburg, Netherlands), and quantified as previously reported by us^23^.

Expression microarray hybridizations were performed using the WT Expression kit (Life Technologies, CA, USA), WT Terminal Labelling and Controls Kit (Affymetrix, CA, USA) and Affymetrix Mouse Gene_1.0ST arrays, which contain 770,317 probe sets representing an estimated 35,556 mouse transcripts. The probed arrays were washed and stained using the GeneChip Hybridization Wash and Stain Kit (Affymetrix, CA, USA) and scanned using the GeneChip Scanner 3000. Images (.dat files) were processed using GeneChip Command Console (Affymetrix, CA, USA) and CEL files imported into Partek Genomics Suite 6.6 (Partek SG, Singapore) for further analysis.

### Gene co-expression network

A gene co-expression network was generated using the Affymetrix Mouse Gene_1.0ST array analyses of thymocyte subsets – a total of 28 microarrays. The Affymetrix CEL files were normalised using RMA background subtraction in Bioconductor and batch effects were removed using the nonparametric CombatR algorithm^46^. Variability of transcripts across all arrays was ranked by standard deviation^47^ and the 1,929 most variable were used for network construction. Application of the WGCNA algorithm in R^48^ generated a weighted gene co-expression network of 1,929 nodes (transcripts) and 10,626 edges (representing significant correlations at a p < 0.02) assigned to 12 significantly co-expressed modules.

### Gene ontology analysis

Gene lists were generally split into those upregulated and those downregulated before being submitted to The Database for Annotation, Visualization and Integrated Discovery (DAVID) Bioinformatics Resources v6.7^49^ for gene ontology analysis of functional annotation clustering against Clusters of Orthologous Groups (COG) Analysis Ontology, Sp Pir Keywords (SP-PIR), Gene Ontology Biological Process (GO-BP), Molecular Function (GO-MF), Cellular Compartment (GO-CC), UniProt Sequence annotation (UP_SEQ_FEATURE), Online Mendelian Inheritance in Man (OMIM_DISEASE), Biological Biochemical Image Database(BBID), and BioCarta Pathways (BIOCARTA).

### In vivo BrdU incorporation assays

NOD.*Va14*^*tg*^ mice received 3 intraperitoneal (i.p.) injections of 1 mg (in 100ul PBS) BrdU (Sigma-Aldrich; B5002) at 12-h intervals for a total of 3 mg/mouse at the indicated time point before harvest. The mice were culled and thymocytes were isolated. One million cells from each sample were pre-incubated for 15 min with unconjugated anti-CD16/32, provided with FITC BrdU flow kit (BD Bioscience, Pharmingen, San Diego, CA, USA) to prevent Fc Receptor binding of labelled antibodies. The cells were then surface stained with antibodies in a U bottom well plate, fixed, permeabilised and intracellular-stained with anti-BrdU FITC following the manufacturer’s protocol. Half a million cells were acquired on a BD Fortessa flow cytometer at a flow rate < 400 events per second.

### First-strand cDNA synthesis

First-strand cDNA was synthesized from 36 to 500 ng of RNA using oligo(dT) primers and Tetro cDNA synthesis kit (Bioline) following manufacturer’s instructions.

### Real-time quantitative PCR

Real-time quantitative PCR (qPCR) was conducted to verify microarray data on independent samples of FACS sorted immature thymic T and iNKT cell subset RNA from NOD.*V*α*14*^*tg*^ and WT mice. All PCRs were carried out on the Rotorgene 6000 (Corbett, Sydney, Australia) and PCR mixes set up using a CAS1200 liquid handling platform (Corbett Robotics, Brisbane, Australia) as done previously^23^. Each 15 µl reaction contained 7.5 µl 2 × SensiFAST SYBR No-ROX mix, 0.6 μL of each primer (2.5 or 10 μM), and 1-6 µl cDNA. PCR conditions included denaturation 50 °C, 2 min, 95 °C, 2 min, then 40 cycles (95 °C, 30 s; 50–56 °C (primer dependent annealing) 30 s; 72 °C, 30 s; 78 °C, 30 s). Fluorescent data were acquired for FAM/SYBR at the 72 °C extension step. A melt curve analysis was conducted by incrementing 1 °C/step from 55 °C until 99 °C. Expression of the gene-of-interest was normalised against *Gapdh*, as microarray expression analyses had shown that this gene was not differentially expressed between NOD.*V*α*14*^*tg*^ and WT mice. The primers used for quantitation were:

*E2f1*F: 5’- GAGGCTGGATCTGGAGACTG-3’.

*E2f1R*: 5’- GAAGCGTTTGGTGGTCAGAT-3’.

*Cdc6*F: 5’- GATCCTGGTTTGCTCTTTGC-3’.

*Cdc6*R: 5’- GAGGCGGCTCTCCTTATTTT-3’.

*Ccne1*F: 5’- TGTTACAGATGGCGCTTGCT-3’.

*Ccne1*R: 5’- GCCAGGACACAATGGTCAGA-3’.

*Cenpm*F: 5’- CGAGCTGAGGGTCCACTTG-3’.

*Cenpm*R: 5’- CAAACACAATCAGGTCAATTCG-3’.

*Rpa2*F: 5’-AGTCCGAGCCCAGCATATTG-3’.

*Rpa2*R: 5’-GTTGGTTGGAGCCTTCTCCG-3’.

*Tcrav7d-3*F: 5’-GTGGTCCTGTGGCTCCAGTTA-3’.

*Tcrav7d-3*R: 5’-CTCTGGGACAATGAGGGATTCTG-3’.

*Gapdh*F : 5’-TGCCGCCTGGAGAAACCTGCCAAGTATG-3’.

*Gapdh*R: 5’-TGGAAGAGTGGGAGTTGCTGTTGAAGT-3’.

Analyses of unknown samples were carried out by comparison to a standard curve for both the gene of interest and the housekeeper. Template standards were prepared by PCR amplification of cDNA from NOD.*Nkrp1*^*b*^ mouse thymi using primers flanking those used for quantitation:

*E2f1*F: 5’-CGATTCTGACGTGCTGCTCT-3’.

*E2f1R*: 5’-CAGTTCAGGTCAACGACACC-3’.

*Cdc6*F: 5’-TTTCGGAAGTTGATGGGAAC-3’.

*Cdc6*R: 5’-ATGAAGATTCTGGGGGCTCT-3’.

*Ccne1*F: 5’-CCTCCAAAGTTGCACCAGTT-3’.

*Ccne1*R: 5’-GTGTGGGTCTGGATGTTGTG-3’.

*Cenpm*F: 5’- CGATGCTCAAAGATGACTGTG-3’.

*Cenpm*R: 5’-CCTGTGACAAGGAAGCACAC-3’.

*Rpa2*F: 5’- GAGAACTGCGACCAGGATGT-3’.

*Rpa2*R: 5’-GCGGAGCTGTCATATCGTCT-3’.

*Tcrav7d-3*F: 5’-TGAGTGTTTCCCTAGTGGTCCT-3’.

*Tcrav7d-3*R: 5’-TGCTGTCTGTACCATGCAAAA-3’.

*Gapdh*F: 5’- ACCACAGTCCATGCCATCACT -3’.

*Gapdh*R: 5’- TCCACCACCCTGTTGCTGTA -3’.

Titrated template standards were processed in parallel with unknown controls.

### Statistical analyses

Qualitative data were compared by Fisher’s Exact Test or contingency table (Chi squared) analysis and quantitative data by Mann Whitney U Test as calculated by GraphPad Prism 6 (La Jolla, CA, USA).

The statistical significance threshold of the microarray study comparing FACS sorted thymic cells from individual NOD.*Va14*^*tg*^ mice was set at a Mann–Whitney U statistic of 0 (i.e. p < 0.001, n = 7; equating to no overlap between groups). We have previously published empiric validations of microarray expression analyses of similar design by using congenic intervals to differentiate “on target” from “off target” differential expression^23,50^.

## Results

### Production and characterisation of NOD.Va14^tg^ mice

Vα14-Jα18 transgenic mice have been previously developed to aid functional and developmental studies of Type I NKT cells^22,44^. Here, we expressed a validated transgenic construct containing an NKT-associated Vα14-Jα18 TCRα chain cDNA (Fig. 1A) on the NOD mouse genetic background, which confers a partial defect in iNKT cell selection and development^5,22,35^. Purified DNA was directly injected into NOD/Lt embryonic pronuclei at the Transgenic Production Facility of the Walter and Eliza Hall Institute, tail tips were genotyped by a PCR spanning from CD4 to TCR sequence to identify transgene incorporation and germ-line transmission (Fig. 1B), and four independent lines were established. Thymic, hepatic and splenic iNKT cell numbers and subsets were assessed by flow cytometric analysis of CD1d-tet^+^TCRβ^+^ lymphocytes. Transgenic lines 1 and 3 had ~ 60–90-fold increases in thymic iNKT cell numbers while lines 2 and 4 had ~ threefold increases; numbers of hepatic iNKT cells were raised ~ fourfold in lines 1 and 3 and by 25–50% in lines 2 and 4; lines 1 and 3 resulted in ~ sevenfold increases in splenic iNKT cell numbers, while lines 2 and 4 had similar numbers to the non-transgenic parental strain (Fig. 1C).

### iNKT cell developmental subsets in NOD.Va14^tg^ mice

**Figure 2:**
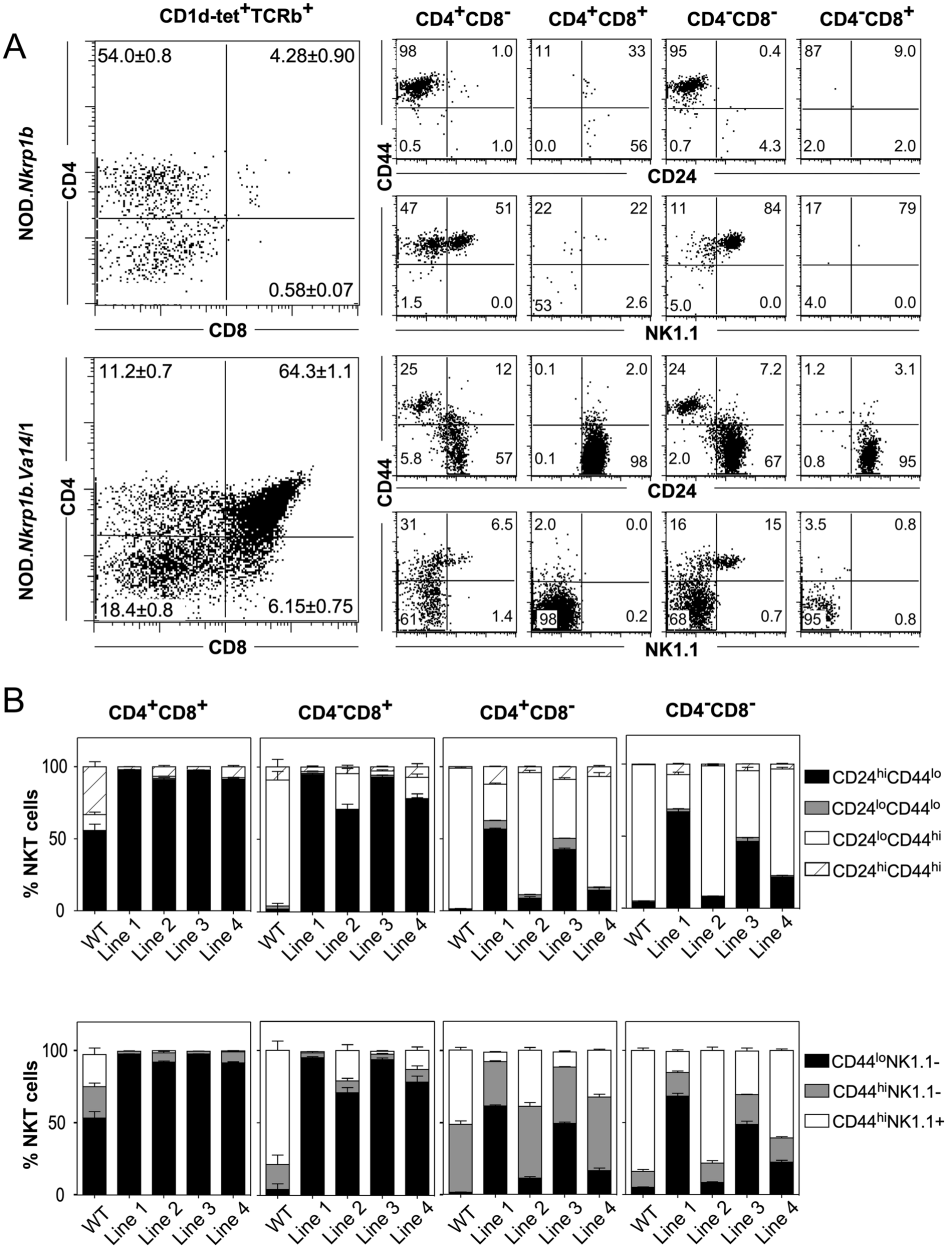
Flow cytometric comparison of thymic NKT cell subsets in WT and NOD.*Va14*^*tg*^ mice. Representative plots are presented of ten female mice/group (**A**) and the relative proportions of the major developmental subsets in each transgenic line compared with those in WT mice presented by bar graph (**B**).

**Figure 3:**
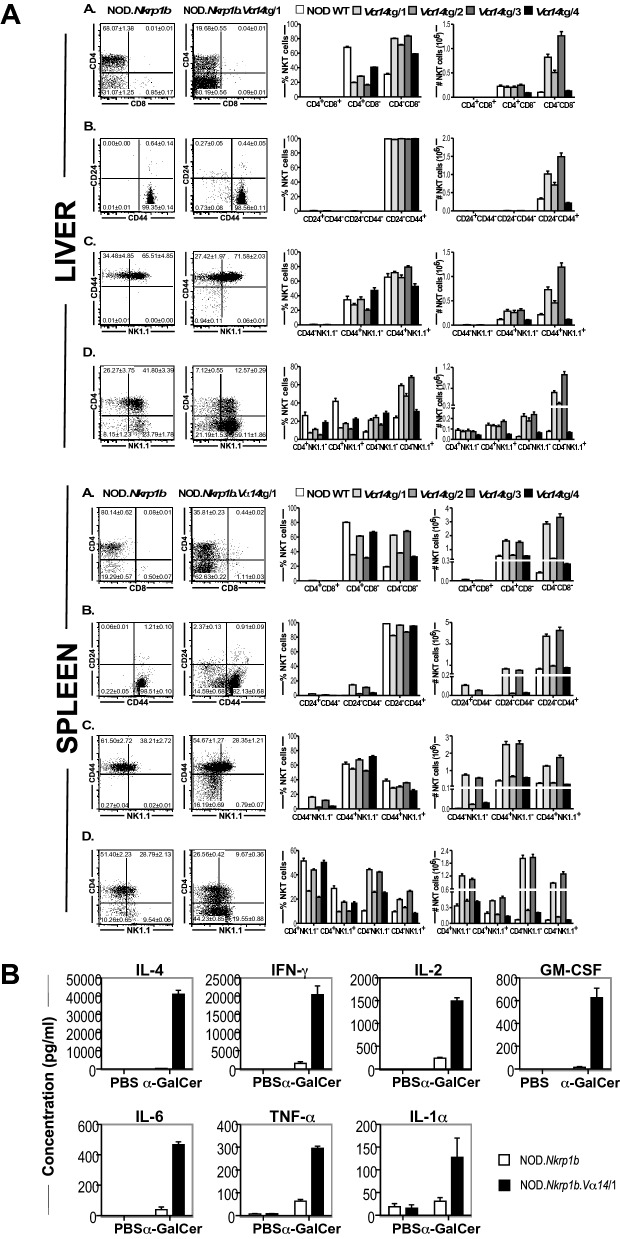
Comparison of hepatic and splenic NKT cell subsets and cytokine production in WT and NOD.*Va14*^*tg*^ mice. A) Representative FACS plots (left panel) show the mean frequencies (± SEM) of NKT cell subsets in total hepatic (Top panels) or splenic (Bottom panels) NKT cells of NOD WT and NOD.*Va14*^*tg*^ mice. (**A**) CD4/CD8 NKT cell subsets. (**B**) CD24/CD44 NKT cell subsets. (**C**) CD44/NK1.1 NKT cell subsets. (**D**) CD4/NK1.1 NKT cell subsets. Histograms (right panel) show the mean frequencies and absolute numbers of hepatic or splenic NKT cell subsets between NOD WT (clear bars) and the four NOD.*Va14*^*tg*^ mouse lines (filled bars). (n = 10 female mice/group. Data are combined from two independent experiments). (**B**) The effect of i.v. α-GalCer administration on cytokine production in NOD.*Va14*^*tg*^ mice shows that Vα14 transgenic expression increased cytokine production. Histograms show the mean concentration (± SEM) of IL-4, IFN-γ, IL-6, IL-2, GM-CSF, TNF and IL-1 in serum of NOD WT mice (clear bars) and NOD.Va14Tg/1 mice (filled bars), 4 h after challenge with α-GalCer (n = 6–10 mice/group).

Both the CD4 SP and DN populations of mature iNKT cells could be identified in the thymi (Fig. 2), livers and spleens (Fig. 3) of WT and NOD.*Va14*^*tg*^ mice.

One of the most striking characteristics of the thymic iNKT cell population in NOD.*Va14*^*tg*^ mice was the presence of increased numbers of DP NKT cells. Their presence in the thymi of previously produced *V*α*14*Tg mice, on either B6^44^ or NOD backgrounds^41^, had not been detected. This may be a consequence of the transgenic system used, a TCRα shuttle vector containing the *V*α*11* endogenous promoter and the Ig enhancer, or it may be possible that the relatively immature NKT cells were inadvertently missed from their analyses due to the detection markers, α/βTCR^+^ and NK1.1^+^ , employed, or alternatively, that they were indeed not increased. Here, the very immature thymic DP NKT cells expressing a CD24^high^ CD44^low^ and NK1.1^−^ phenotype, were identified using TCRβ and CD1d-tetramer, along with stage-specific markers. This therefore, represents the first mouse model to produce large numbers of immature DP NKT cells. This population constituted between 35 and 65% of all thymic CD1d-tet^+^TCRβ^+^ NKT cells in the various lines of NOD.*Va14*^*tg*^ mice, but < 5% of thymic iNKT cells in WT NOD mice. The DP NKT cells were predominantly CD24^hi^CD44^lo^NK1.1^−^, consistent with the phenotype of the earliest identifiable iNKT cells. Another population of iNKT cells also lay within the CD8 quadrant gate of our analysis of NOD.*Va14*^*tg*^ mice; comparison with Fig. 2A of Gapin et al.^29^ suggested that these correlate with the DP^dull^ population previously identified as immediately post-selection iNKT cells. Consistent with this, these cells also expressed a relatively immature phenotype: CD24^hi^CD44^lo^NK1.1^−^.

In contrast to thymic NKT cells, virtually all (> 99%) iNKT cells in the livers and spleens of NOD.*Va14*^*tg*^ mice were either CD4^+^ or DN. In both peripheral organs of NOD.*Va14*^*tg*^ mice, the proportions of the two major iNKT cell subsets that express the relatively mature CD24^lo^CD44^hi^ phenotype were similar to WT, as were the proportions of CD44^hi^ iNKT cells of either subset that expressed NK1.1 (Fig. 3A). These peripheral iNKT cells were functional and responded to in vivo stimulation with α-GalCer by robust cytokine production (Fig. 3B). These results suggest that although expression of the *Va14* transgene on this genetic background has resulted in increased numbers of mature peripheral NKT cells, the majority of immature iNKT cells fail to mature and leave the thymus.

### Transcriptional analysis of immature iNKT cells in NOD.Va14^tg^ mice

The presence of large numbers of DP^hi^CD24^hi^CD44^lo^NK1.1^−^, CD1d-tet^+^TCRβ^+^ cells in the thymus and their absence in the periphery, suggested that these cells represent a very immature—possibly preselection—population of iNKT cells. This gave us the opportunity to compare the transcriptional profiles of DP CD24^hi^CD44^lo^NK1.1^−^CD1d-tet^+^TCRβ^+^ cells (i.e. very immature iNKT cells) with those of somewhat more mature iNKT cell subsets in order to identify the transcriptional transitions associated with positive selection and lineage commitment in NKT cells.

**Figure 4:**
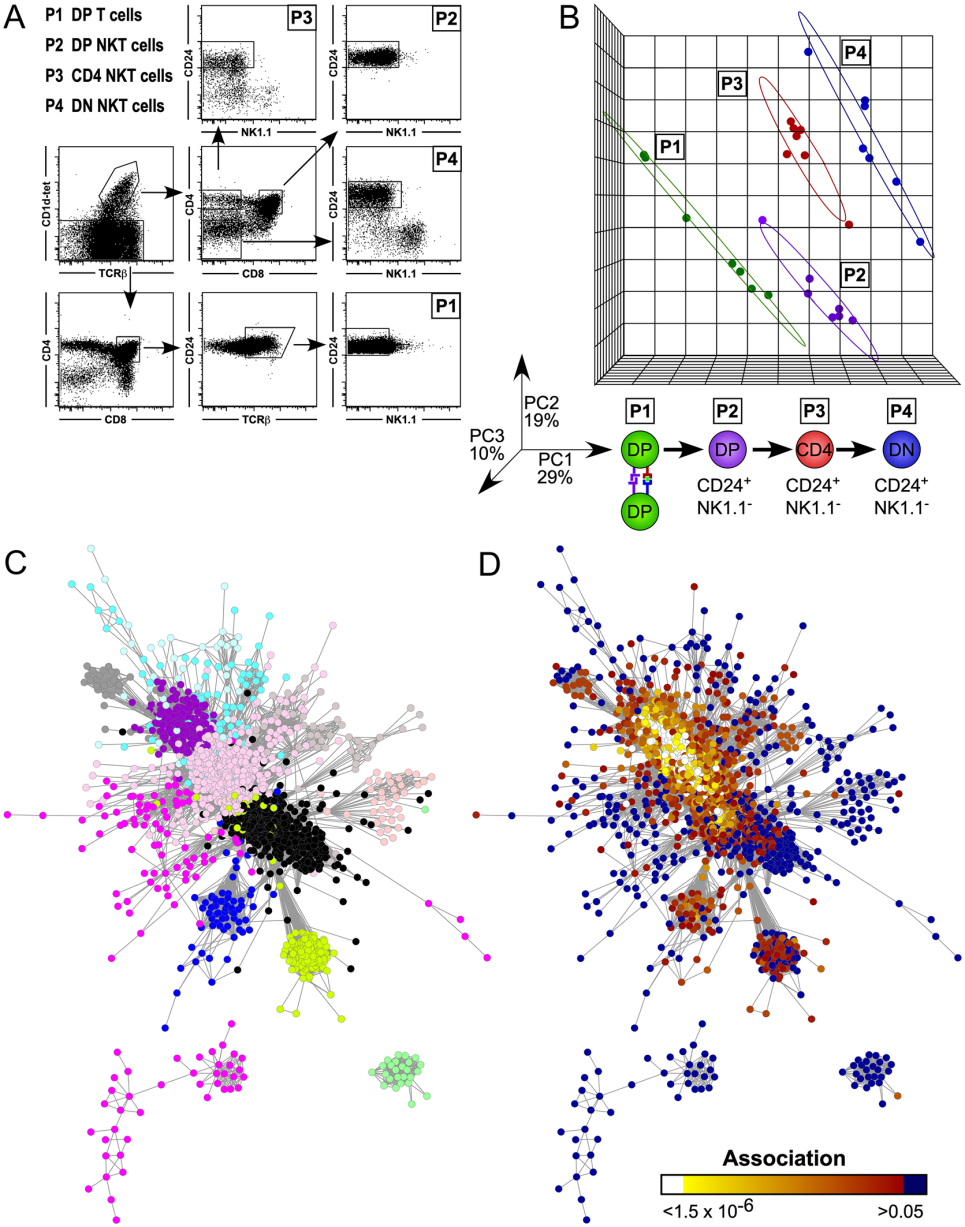
Microarray transcriptional comparison of FACS sorted cells. Microarray transcriptional comparison of FACS sorted thymic DP^hi^ CD24^hi^ NK1.1^−^ CD1d-tet^−^TCRβ^+^ (Population (P) 1 (P1)) cells, DP^hi^ CD24^hi^ CD44^lo^ NK1.1^−^ CD1d-tet^+^TCRβ^+^ (P2) cells, CD4^+^CD8^−^CD24^hi^ NK1.1^−^CD1d-tet^+^TCRβ^+^ (P3) cells and CD4^−^CD8^−^ CD24^−^ NK1.1^+^CD1d-tet^+^TCRβ^+^ (P4) cells from individual NOD.*Va14*^*tg*^ /1 mice (n = 7). (**A**) Gating strategy for sorting of populations. (**B**) Principal component (PC) analysis of transcript expression. The three largest PC explain > 58% of variation; samples from each of the four cell types cluster together into groups, and the groups are distributed separately across PC1 (X axis), which explains > 29% of variation. A graphic illustrating the proposed NKT cell maturation pathway is provided for comparison. (**C**) Undirected, weighted transcriptional regulatory network generated from thymic NKT cell subsets P1–P4 (n = 7/population), consisting of 1,929 transcripts (nodes) and 10,626 pair-wise correlations (edges) across twelve co-regulated clusters (modules), named: Black (587 nodes), Cyan (71), GreenYellow (177), Grey (32), LightCyan (42), LightGreen (26), Magenta (127), MidnightBlue (68), Pink (525), Purple (188), Salmon (37) and Tan (49). (**D**) Heat map encoding of student’s t-test p values generated by pair-wise comparisons of transcript abundance across Transition 1 (P1 to P2) mapped onto the transcriptional regulatory network illustrated in panel C. (The WGCN depicted in (**C**,**D**) were visualised using a heat map in Cytoscape 3.8.0; https://github.com/cytoscape/).

Thymocytes from transgenic Line 1 mice (NOD.*Va14*^*tg*^/1) were subjected to FACS sorting to isolate four subsets; One DP-CD1d tet^−^ population and three CD24^hi^ CD1d tet^+^ (immature) subsets: DP^hi^CD24^hi^NK1.1^−^CD1d-tet^−^TCRβ^+^ cells (immature DP conventional T cells), DP CD24^hi^CD44^lo^NK1.1^−^CD1d-tet^+^TCRβ^+^ cells (DP NKT cells), CD4^+^CD8^−^CD24^hi^ NK1.1^−^CD1d-tet^+^TCRβ^+^ cells (CD4 NKT cells) and CD4^−^CD8^−^CD24^hi^NK1.1^−^CD1d-tet^+^TCRβ^+^ cells (DN NKT cells). RNA was isolated for microarray analysis with Affymetrix Mouse Gene_1.0 ST arrays (n = 7/grp; Fig. 4A). In our experience of microarray expression analyses of mouse thymocytes, the use of seven replicates and a significance threshold of a Mann–Whitney U (MWU) statistic of zero (i.e. no overlap between groups) provides robust and reliable identification of differentially expressed transcripts^23,24,50^.

CEL files from all 28 samples were imported into Partek Genomics Suite 6.6 (Partek SG, Singapore) where a Principal Component (PC) analysis plot was generated based on the non-normalised data (as present in the *Gene counts* data mode). Here, samples are represented as dots and distance between each other reflects similar expression patterns of a large number of genes, while those with large gene numbers with different expression patterns are further apart. Our analysis indicated that the three largest PCs explained > 58% of variation across all our samples, and that they grouped into 4 groups. When coloured using the first categorical variable, the Cell subtype, our samples from each of the four cell types were shown to cluster together into their individual groups, and the groups were distributed evenly across PC1 (X axis, which explained > 29% of variation) in the order DP T cells → DP NKT cells → CD4 NKT cells → DN NKT cells (Fig. 4B). The transcriptional progression of these immature iNKT cell subsets therefore mirrors the postulated developmental progression previously described^51,52^. Of particular interest is the possibility that our analysis of DP NKT cells might identify the transcriptional events associated with Control Point 1 of iNKT cell development, which may be associated with positive selection^51^.

### Weighted gene co-expression network

A transcriptional regulatory network was constructed using the Weighted Gene Correlation Network Analysis (WGCNA) algorithm in R^48^, representing the expression data from 35,557 transcripts from each of the 28 samples (7 replicates × 4 populations; GSE106720). The resulting network contained 1,929 nodes (transcripts) in twelve modules (co-expressed transcript clusters) with 10,626 edges (pair-wise significant correlations; Fig. 4C). The network was then analysed as three progressive transitions: Transition 1) DP T cells → DP NKT cells; Transition 2) DP NKT cells → CD4 NKT cells; Transition 3) CD4 NKT cells → DN NKT cells. For each transition, uncorrected Student’s t-Test p values of pair-wise comparisons of each transcript (GSE106720) were mapped onto the nodes within the network and visualised using a heat map in Cytoscape 3^53^ (Fig. 4D). Highly differentially expressed (HDE) transcripts were defined as significant after Bonferroni p value correction (p < 1.5 × 10^–6^).

### Transition 1: Modulation of TCR signaling

Across transition one, 311 transcripts were identified in the network as being HDE. Of these, 116 were in the module named “Pink” (of 525 nodes) and 48 were in the Purple module (of 188 nodes; p < 0.0001 χ^2^ contingency; Fig. 4D). Gene Ontology Analysis was performed on the transcripts within the Pink module in The Database for Annotation, Visualization and Integrated Discovery (DAVID) Bioinformatics Resources v6.7^49^. The top ranked Category (SP_PIR_KEYWORDS) obtained for the transcripts in the Pink module was oxidative phosphorylation with a Bonferroni-corrected p value < 4.1 × 10^–6^. Within this category were genes encoding several components of the mitochondrial electron transport chain, NADH Dehydrogenase (*Nd1*, *Nd2*, *Nd4l*, *Nd5*), Cytochrome C Oxidase (*Cox1*, *Cox2*, *Cox3*) and ATP Synthase 6 (*Atp6*), all of which were downregulated across Transition 1. This finding is consistent with the intracellular metabolic diversion of small carbon chains away from oxidative phosphorylation to fatty acid synthesis, as proposed by Warburg to be indicative of increased cell proliferation^54^.

**Figure 5:**
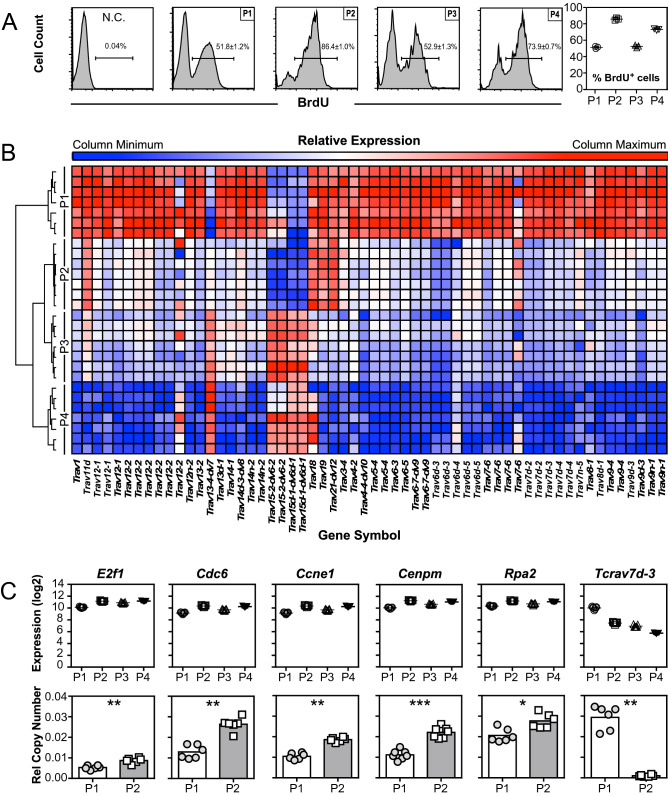
Generation of undirected, weighted transcriptional regulatory network from thymic NKT cell subsets. (**A**) Representative histograms and dot plot displaying proportion of BrdU positive cells among DP T (P1), DP NKT (P2), CD4 NKT (P3) and DN NKT (P4) cells compared to a non-injected control (NC). (**B**) Heat map of endogenous TCR alpha chain loci expression determined by microarray of the four different cell types across the three transitions as visualised in Cytoscape 3.8.0 (https://github.com/cytoscape/). (**C**) Expression profiles of genes associated with the cell cycle and cell division. Gene expression as determined by microarray (top row; n = 7/grp) and confirmed by qPCR on an independent cohort (bottom row; n = 7/grp).

Evidence of increased proliferation across Transition 1 was therefore sought by in vivo Bromodeoxyuridine (BrdU) uptake. NOD.*Va14tg* mice received three intraperitoneal (i.p.) injections of 1 mg BrdU at 12-h intervals, the mice were culled and thymocytes were examined by flow cytometry (Fig. 5A). Transition 1 was associated with a 55% increase in cells taking up BrdU in the 36-h labelling period (p < 0.0002; Mann Whitney U Test; n = 8), confirming increased proliferation.

Transcriptional evidence was sought to support the hypothesis that the increase in proliferation across Transition 1 was due to TCR signalling. The expression levels of all 35,556 transcripts across Transition 1 were compared pairwise by Mann–Whitney U (MWU) Test, and those with a U statistic of zero (i.e. no overlap between groups) shortlisted for ranking by t-Test (i.e. transcripts for which the means of the groups were separated by largest multiples of SEM were prioritised). Across this transition, 5,565 transcripts generated a Mann–Whitney U score of zero, and of these, 1,195 were HDE (p < 1.4 × 10^–6^, by t-Test). This gene list was split into transcripts upregulated (624 transcripts), and those downregulated (570), before being submitted to DAVID for functional annotation clustering.

The top downregulated annotation cluster (Enrichment Score 7.3) contained the annotation categories Lymphocyte Activation (GO:0046649), Leukocyte Differentiation (GO:0002521) and T Cell Activation with Bonferroni corrected p values between 10^–6^ and 10^–4^. Remarkably, genes encoding almost all the proximal components of the TCR signalling cascade were significantly down regulated (U stat = 0): *Lck* (p < 6.6 × 10^–8^; student’s t-test), *Fyn* (1.4 × 10^–7^), *Zap70* (5.4 × 10^–5^), *Vav1* (1.3 × 10^–6^) and *Plcg1* (1.2 × 10^–10^). Although not previously reported, the effects of this transition would be akin to other mechanisms of TCR tuning, such as down regulation of the TCR and CD4/CD8 co-receptors, regulation of accessory molecules such as CD5, CD2 and CD28, and phosphorylation of SHP-1^55^, suggesting the possibility that the signalling pathway was modulated or “tuned” subsequent to TCR ligation and successful signalling.

If the proliferation observed across Transition 1 was due to successful TCR signalling, then allelic exclusion of endogenous alpha chain loci should have occurred. Consistent with this, TCR alpha chains dominated the most significantly differentially expressed transcripts, constituting ten of the top 20 most significant. Highly significant down-regulated TCR variable-alpha gene segments included: *Tcra-V22.1, Trav8d-1, Trav7d-4, Trav12-2, Trav9-4, Trav6-5, Trav9n-1, Trav6-3, Trav7d-3, Trav9d-3, Trav13d-1, Trav7d-2, Trav6-7-dv9, Trav7-6, Trav6d-5, Trav9-4, Trav13-2, Trav12-2, Trav12n-2, Trav14-1, Trav7-6, Trav5-4, Trav6d-3, Trav1, Trav14n-2, Trav12-2, Trav12-1, Trav7d-4, Trav4-4-dv10, Trav9d-3, Trav7n-5, Trav14d-3-dv8, Trav8-1* and *Trav12-1* (all U = 0; p < 1.4 × 10^–6^, t-Test; Fig. 5B). The dearth of expression of the great majority of non-NKT cell associated Vα chains is consistent with successful signalling of the transgene-encoded TCR initiating the mechanism for allelic exclusion of competing TCRs.

The possibility of increased proliferation in DP NKT cells was also supported by the up-regulated expression of genes associated with the cell cycle and cell division. A large number of genes encoding proteins involved in DNA replication, components of centromeres and the E2F signalling pathway, were significantly up-regulated by immature DP NKT cells compared to immature DP T cells. *E2f1*, which encodes the E2F1 transcription factor, was highly significantly up-regulated across Transition 1 (p < 9.1 × 10^–11^, student’s t-test; Fig. 5C). Consistent with increased *E2f1* expression, E2F1-regulated genes encoding proteins critical for S phase entry^56^ were also up-regulated, such as *Cdc6* (p < 1.4 × 10^–9^, student’s t-test) and *Ccne1* (p < 8.7 × 10^–8^, student’s t-test; Fig. 5C). Furthermore, consistent with E2F1 up-regulation driving cell proliferation, genes encoding proteins critical for spindle formation and chromosome segregation (such as *Cenpm)*^57^ and DNA replication (such as *Rpa2*)^58^ were also up-regulated (p < 3.7 × 10^–11^; and p < 4.6 × 10^–10^ respectively; Fig. 5C).

In summary, the combination of activation, proliferation, TCR tuning and allelic exclusion combine to provide compelling evidence that TCR signaling occurred across Transition 1.

### Transition 2: Lineage Commitment

In Transition 2, from DP NKT cells—> CD4 NKT cells, 6,791 transcripts generated a Mann–Whitney U score of zero. Of these, 2,143 were HDE (student’s t-test p < 1.4 × 10^–6^), 125 of which mapped to the Weighted Gene Co-expression Network, with 33 of them in the module named “Pink” and 85 in the module named “Purple”.

The full HDE gene list was split into transcripts upregulated (1,216 transcripts), and those downregulated (930 transcripts), before submitting it to DAVID for annotation.

**Figure 6:**
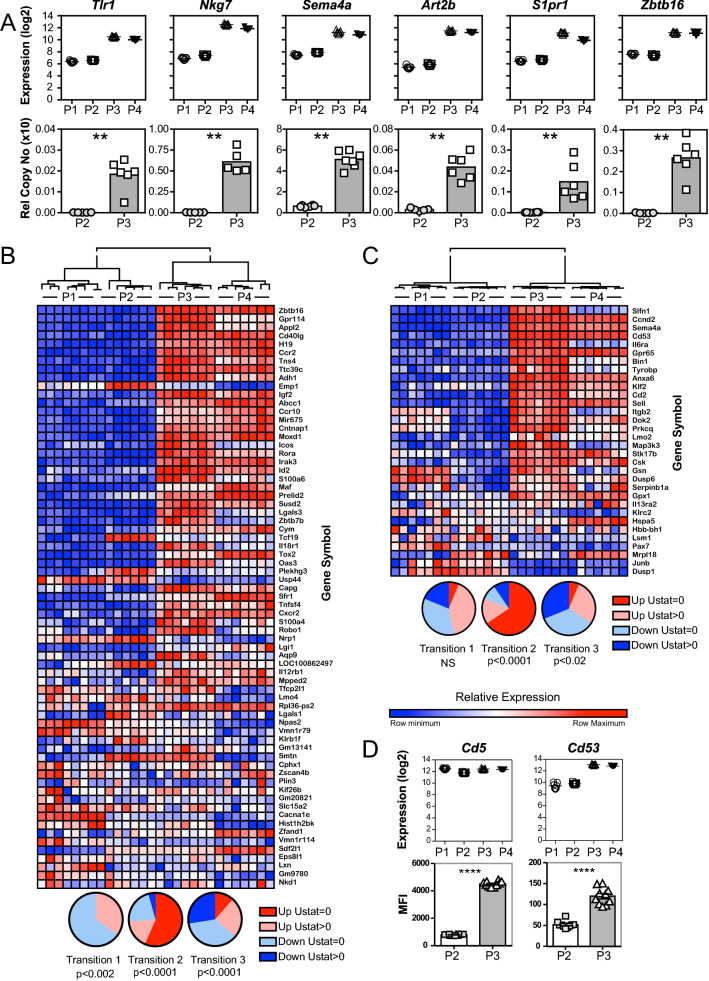
Gene expression profiles. (**A**) Expression profiles of genes related to lineage commitment across Transition 2. Gene expression as determined by microarray (top row; n = 7/grp) and confirmation by qPCR on an independent cohort (bottom row; n = 7/grp). (**B**,**C**) Heat maps showing expression profiles of the different cell types across the three transitions for the 69 transcripts, reported by Savage et al.^46^ to be modulated by Promyelocytic Leukaemia Zinc Finger (PLZF) (**B**) and the 32 transcripts, reported by Huang et al.^47^ to involve in positive selection of conventional T cells (**C**). Pie charts show proportion of transcripts up- and down-regulated across the three transitions (up-regulated and U statistic = 0, up-regulated and U statistic > 0, down-regulated and U statistic = 0, and down-regulated and U statistic > 0) (Heat maps, hierarchical clustering and pie charts depicted in B and C were prepared using Cytoscape 3.8.0 https://github.com/cytoscape/)) (**D**) Expression profiles of leukocyte differentiation markers, *Cd5* and *Cd53* as determined by microarray (top row) and protein levels confirmed by flow cytometry on an independent cohort (bottom row; n = 10 female mice per group; Mann–Whitney U test, ****p < 0.0001).

The top upregulated annotation cluster (Enrichment Score 9.6) was dominated by integral plasma membrane proteins and contained the annotation categories (SP_PIR_KEYWORDS) Membrane (representing 40 of all upregulated HDE genes; Bonferroni corrected p < 1.6 × 10^–17^) and Glycoprotein (corrected p < 1.1 × 10^–14^). Several functional families of integral plasma membrane proteins dominated the gene list, including genes encoding Toll-like receptors (TLR1, 3, 6, 12), receptors for cytokines (IL-1, 2, 6, 7, 10, 18, 21, 27; IFN- α, TGF-β and TNFR superfamily members 1b, 9, 18 and 26), receptors for chemokines (CCR2, 4, 7, 8, 10; CXCR2 and 6), G protein coupled receptors (18, 65, 68, 83, 97, 114, 171, C5B), integrins (alpha 2, 4, E, L, V and FG-GAP repeat containing 3; beta 2, 3 and 7) and leukocyte differentiation markers (CD2, 5, 7, 37, 38, 40LG, 44, 48, 49, 50, 59A, 79B, 82, 101, 160, 226, 274: Ly6A, C1, 6G and Ly9). The difference in protein expression levels were confirmed by QPCR (Fig. 6A) or flow cytometry (Fig. 6D) for several of the transcripts.

Of the upregulated HDE transcripts, amongst the most strongly differentially expressed (U stat = 0) were genes associated with the immunomodulatory and innate-like characteristics of iNKT cells, such as: *Tlr1* (p < 1.1 × 10^–16^, t-Test; Fold Change (FC) 15) which encodes Toll-Like Receptor 1 and plays a fundamental role in pathogen recognition and activation of innate immunity; *Nkg7* (p < 3.8 × 10^–15^; FC 36) which encodes Natural Killer Cell Granule Protein 7, which is associated with the cell mediated cytotoxic synapse; *Sema4a* (p < 9.6 × 10^–10^; FC 10), which encodes Semaphorin 4A, a type I integral membrane protein required for Th1 deviation and T-bet expression in T cells; *Art2b* (p < 1.5 × 10^–14^; FC 49) which encodes ADP-ribosyltransferase 2b that mediates apoptotic deletion of T-cell subsets^59^, particularly CD4^+^ NKT cells^60^; *S1pr1* (p < 5 × 10^–16^; FC 22), which encodes Sphingosine-1-Phosphate Receptor 1, which plays an important role in lymphocyte egress from lymphoid tissues; and *Zbtb16* (p < 6.5 × 10^–14^; FC 14), which encodes Promyelocytic Leukaemia Zinc Finger (PLZF), a transcription factor that drives differentiation into iNKT cells and human MR1-specific MAIT cells (Fig. 6A and Supplementary Table 1). All of these strongly upregulated transcripts lay within the PURPLE module, and had been identified in the network analysis. These data indicate that a wide range of iNKT cell-associated surface receptors are upregulated at this transition and confirm the finding by Savage et al.^61^ and Cohen et al.^62^ that *PLZF* expression in iNKT cells is upregulated between Stage 0 and Stage 1 in development.

The strong upregulation of such a wide range of functional lymphocyte-associated, surface-expressed, integral membrane proteins suggests that Transition 2 is associated with iNKT cell lineage commitment. Particularly indicative is the upregulation of *Zbtb16*, which is responsible for driving the innate-like differentiation of iNKT cells^63^. To test whether the upregulation of *Zbtb16* across Transition 2 was sufficient to modulate PLZF target gene expression, we examined the transcript levels of the 69 genes identified by Savage et al.^61^ as being dependent on PLZF expression by comparison of genome-wide transcription in PLZF-deficient *Luxoid* mutant with that in WT mice. Consistent with PLZF expression playing a significant role in Transition 2 of iNKT cell development, 42 of the 69 genes identified by Savage et al. had a U statistic of zero across the transition (p < 0.0001; Chi-square contingency table), the vast majority (39/42) of which were upregulated (Fig. 6B). In addition, members of the killer-cell lectin-like receptor group-B receptor ligands, variably known as NK1/NK1.1, were upregulated across this transition , including *Klrb1a* (1.8 × 10^–13^; FC 3.63) and *Klrb1c*, the major target of anti-NK1.1 monoclonal antibody (PK136) binding known to identify NK cells from B6 and SLJ mice (P < 1.81 × 10^–6^; FC 1.63).

In summary, the coordinated expression of the innate-like lymphocyte-associated transcription factor PLZF and the subsequent upregulation of a wide range of cell-surface functional receptors associated with iNKT cell immunobiology combine to provide evidence that iNKT cell lineage commitment occurred between Population 2 and Population 3, across Transition 2, and confirms previous reports by Savage et al.^61^ and Cohen et al.^62^ that this occurs by stage 1 in development.

### Transition 2: Selection

The association of modulation or tuning of iNKT cell TCR signalling with Transition 1 and lineage commitment with Transition 2, raised the issue of the timing of iNKT cell selection, which presumably could not occur later than Transition 2. Huang et al.^64^ published a custom microarray analysis of transcriptional changes during T cell selection by comparing transcripts between DP thymocytes from MHC-deficient mice (C57BL/6.*B2m*^−*/*−^*.Ab*^−*/*−^*.E*^*null*^) with those from positively selecting TCR transgenic mice (5CC7, from an A^b^-restricted CD4 cell specific for pigeon cytochrome c; and either F5, originated from a D^b^ restricted CD8 cell specific for influenza virus nucleoprotein; or P14, from a D^b^-restricted CD8 cell specific for lymphocytic choriomeningitis virus glycoprotein). We identified equivalent Affymetrix transcript cluster IDs for 32 of the 44 transcripts reported by Huang et al. as being up-regulated by T cell selection; of these, 8 had a U statistic of zero across Transition 1 (NS, Chi-square contingency table), 24 did so across Transition 2 (p < 0.0001, and 12 did so across Transition 3 (p < 0.02). At Transition 2, all but three transcripts with a U Statistic of 0 were upregulated; in total 26 of the 32 transcripts were upregulated across this transition (Fig. 6C).

**Figure 7:**
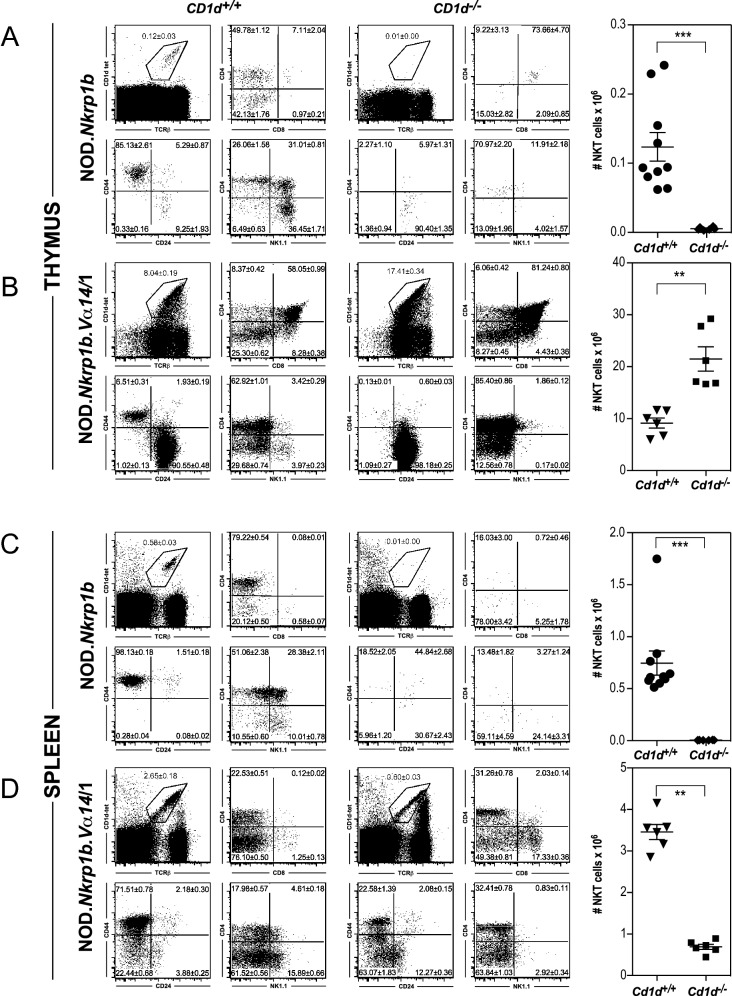
Flow cytometric analysis of the effects of targeted deletion of CD1d in WT NOD mice and NOD.*Va14*^*tg*^ mice. Thymic (**A**,**B**) and splenic (**C**,**D**) NKT cell numbers and subsets in NOD WT (**A**, **C**) and NOD.*Va14*^*tg*^ /1 (**B**,**D**) mice. **P < 0.01; ***P < 0.001; Mann–Whitney U Test; n = 6–10 mice/group.

These data are consistent with iNKT cell selection continuing across Transition 2. To test this, the effect of failed positive selection on iNKT cells in NOD.*Va14*^*tg*^ mice was examined in NOD.*Va14tg.Cd1d*^−*/*−^ mice lacking the CD1d NKT cell selecting ligand in comparison with WT NOD mice and NOD.*Va14*^*tg*^ mice. In WT NOD mice, targeted deletion of CD1d resulted in the loss of almost all CD1d-tet-staining cells, both in the thymus and the periphery (Fig. 7A,C). In contrast, although NOD.*Va14*^*tg*^.*Cd1d*^−*/*−^ mice had greatly reduced numbers of peripheral iNKT cells (Fig. 7D), the numbers of thymic CD1d-tet-staining cells were more than doubled, with the proportion of iNKT cells that were DP rising from ~ 60% to over 80% (p < 0.005; Mann–Whitney U Test; n = 6–10; Fig. 7B) and the vast majority (> 98%) expressing the Population 2 (DP CD24^hi^ CD44^lo^ NK1.1^−^) phenotype.

With regard to absolute numbers, NOD.*Va14*^*tg*^ mice had 8.30 ± 0.56 × 10^6^ (mean ± SEM) Population 2 iNKT cells, while NOD.*Va14*^*tg*^.*Cd1d*^−*/*−^ mice had a > twofold increase to 21.44 ± 1.87 × 10^6^ cells (p < 0.005; Mann–Whitney U Test). In contrast, numbers of mature thymic (CD24^lo^CD44^hi^NK1.1^−^) NKT cells fell more than 20-fold from 5.99 ± 0.73 × 10^5^ in NOD.*Va14*^*tg*^ mice to 0.29 ± 0.06 × 10^5^ in NOD.*Va14*^*tg*^.*Cd1d*^−*/*−^ mice (p < 0.005; Mann–Whitney U Test). These changes are consistent with maturational arrest between these two stages in *Cd1d*^−*/*−^ mice resulting in a backlog of DP^hi^CD24^hi^CD44^lo^ pre-selection iNKT cells and a failure of the lineage to develop past the maturational step associated with positive selection.

As the CD1d-deficient mouse strain used here^43^ disrupts only the *CD1d1* gene, and, unlike the B6 strain who lack CD1D2 protein expression (due to a frame-shift mutation at the beginning of the fourth exon encoding the α3 domain), NOD mice express high levels of CD1D1 and CD1D2 thymic transcripts at a ratio close to 1:1^65^, it was therefore possible that CD1D2 molecules impacted iNKT cell selection and function in this strain, perhaps by presenting a different repertoire of self-antigens than CD1D1^66^. While NKT cells were undetected in the thymi of the targeted deletion NOD strain, it was possible that the presence of the transgene may have impacted their detection in NOD.*Va14*^*tg*^.*Cd1d*^−*/*−^ mice. In order to confirm that the maturational arrest of immature DP NKT cells in NOD. *Va14*^*tg*^ mice was a consequence of the absence of CD1d endogenous ligand, and not due to the NOD background strain, NKT cells were also examined in B6. *Va14*^*tg*^.*Cd1d*^−*/*−^ mice. Thymic NKT cells were more than quadrupled, increasing from 7.8% of total thymocytes of B6.*Va14*^*tg*^ mice to almost 21% in B6.*Va14*^*tg*^.*Cd1d*^−*/*−^ strain, concordant with very few peripheral NKT cells (p < 0.005; Mann–Whitney U Test; n = 5–7). In contrast, targeted deletion of CD1d in B6 WT mice, resulted in the loss of almost all NKT cells in the thymi, livers and spleens. Similar to that found in NOD.*Va14*^*tg*^.*Cd1d*^−*/*−^ mice, the increase in thymic NKT cells was largely attributed by the accumulation of immature DP NKT cells, rising from 48% of total thymocytes in B6.*Va14*^*tg*^ mice to over 85% in B6.*Va14*^*tg*^.*Cd1d*^−*/*−^ mice (p < 0.005; Mann–Whitney U Test; n = 5–7). Absolute numbers of thymic DP NKT cells increased more than tenfold, from 1.2 × 10^6^ in B6. *Va14*^*tg*^ mice to 13.6 × 10^6^ cells in B6. *Va14*^*tg*^.*Cd1d*^−*/*−^ mice, while mature CD24^lo^ CD44^hi^ NKT cells were severely diminished.

This accumulation of immature DP NKT cells may therefore be a consequence of early TCRα expression due to the presence of the transgene^67^ and may be dependent on Vβ usage^68^. Indeed, it has been previously reported that in a few TCRβ rearranging cells, TCRα proteins are expressed so early that they mimic the pre-TCRα chain with regard to induction of cell maturation as well as allelic exclusion^69^. The NOD.*V*α*14*^tg^/1mice exhibited the same characteristic of TCR Vβ usage as NKT cells from WT mice, however, 70–80% of CD44^−^ NKT cells and ~ 38% of CD44^+^ NKT cells in the thymus of NOD. *Va14*^tg^ mice used TCR Vβ chains other than Vβ8, Vβ7 or Vβ2, exceeding by far the frequency detected in the thymi of WT NOD mice (~ 0%; data not shown). These findings suggest that the choice of TCRβ chain affects the probability that a α-GalCer/mCD1d tetramer-binding thymocyte will transition from a CD44^−^ to a CD44^+^ phenotype. These results reflect the findings of Bedel et al.^70^ who constructed an unusual TCR-β chain, that when paired with the canonical Vα14-Jα18 iNKT TCR-α chain, showed increased affinity of the αβ TCR for the antigen/CD1d complex, regardless of the antigen involved. iNKT cell precursors that expressed the high-affinity TCR for “self” in these mice did not fully mature, thus providing direct in vivo evidence that iNKT cells, like their “conventional” T-cell counterparts, are subject to clonal deletion during development. Unexpectedly, they also found that a small fraction of cells with iNKT cell properties, avoided negative selection in these mice, just as in our analyses. Klibi and Benlagha^71^ also reported that NKT cell migration out the thymus can occur in the absence of CD1 expression, but that it, and other key components necessary for NKT positive selection, are required for maturation in peripheral organs.

These results are consistent with the previous findings that iNKT cell positive selection and lineage commitment occur at the DP stage^29,71–73^.

### Effect of enhanced negative selection on iNKT cells in NOD.Va14^tg^ mice

Negative selection in the thymus is a process resulting in clonal deletion (apoptosis) of thymocytes with high avidity for the endogenous ligands of their TCR. As the T precursor frequency for any given antigen is generally low in WT animals, in vivo models of negative selection either involve administration of anti-TCR/CD3 antibodies^74^, responses to endogenous or administered superantigens that ligate whole families of TCR Vβ chains^75,76^, or TCR transgenic mice^77^. Although α-GalCer is technically not a superantigen, it is a strong NKT cell glycolipid antigen capable of activating the vast majority of Vα14-Jα18 expressing NKT cells following its presentation in the context of CD1d^78^, and is capable of causing their negative selection if administered while they transition through a susceptible developmental window before they exit the thymus^79^. Pellici et al.^79^ showed that CD4^+^CD1d-tet^+^Nk1.1^−^ cells (Population 3 cells in our analysis, which were the first stage of iNKT cells that they could clearly detect) were already beyond the “negative-selection” window.

**Figure 8:**
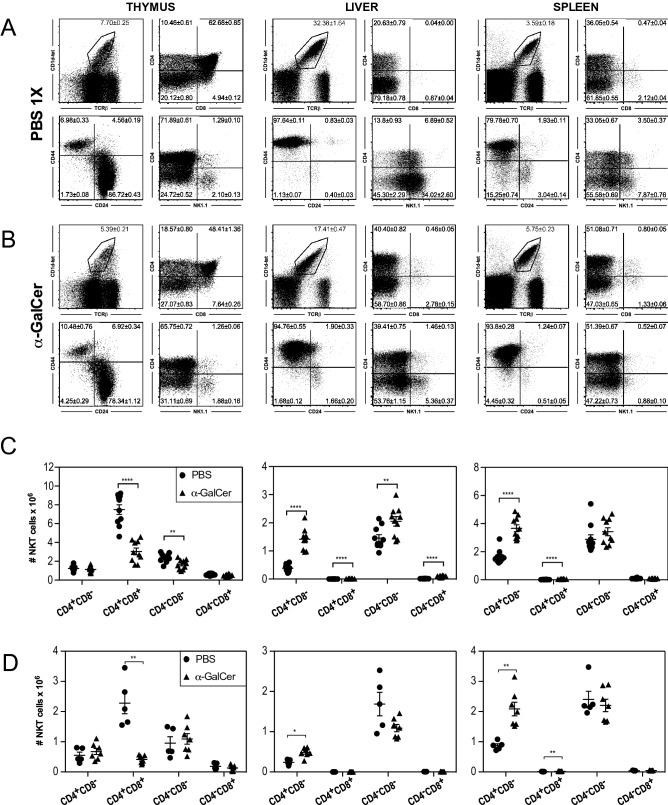
Effect of i.v. or intra-thymic injection of α-GalCer on NKT cells in NOD.*Va14*^*tg*^ mice. Flow cytometric comparison of the effects of i.v. injection with PBS (**A**) or α-GalCer (**B**) on thymic, hepatic and splenic NKT cell proportions, subsets (**A**,**B**) and absolute numbers (**C**) in NOD.*Va14*^*tg*^ /1 mice (**P < 0.01; ****P < 0.0001; Mann–Whitney U Test; n = 10 mice/group). (**D**) Flow cytometric comparison of the effects of intrathymic injection of α-GalCer on absolute numbers in NOD.*Va14*^*tg*^ mice compared to untreated mice. (*P < 0.05; **P < 0.01; Mann–Whitney U Test; n = 5–7 female mice/group). Filled circles represent PBS treated mice; filled triangles represent α-GalCer-treated mice.

The effects of negative selection on Population 2 (DP^hi^CD24^hi^CD44^lo^NK1.1^−^) NKT cells in the thymi of NOD.*Va14*^*tg*^ mice was examined by i.v. injection of α-GalCer, and subsequent flow cytometric analysis of numbers and subsets of iNKT cells (Fig. 8). Within 40 h of injection, total thymocytes had only slightly decreased from 1.55 ± 0.10 × 10^8^ (mean ± SEM) to 1.15 ± 0.11 × 10^8^ cells (p < 0.05; Mann–Whitney U Test; n = 10) while the proportion of (CD1d-tet ^+^TCRβ^+^) NKT cells reduced from 7.75% of total thymocytes in PBS-control mice to 5.4% in α-GalCer injected mice, halving the numbers of NKT cells from 11.9 ± 0.7 × 10^6^ to 5.4 ± 0.2 × 10^6^ cells (p < 0.0001). The majority of the reduction in thymic iNKT cells was contributed by a 60% reduction in numbers of the DP subset, which fell from 62.7 ± 0.9% to 48.4 ± 1.4% of iNKT cells (7.5 ± 0.5 × 10^6^ to 3.1 ± 0.4 × 10^6^ cells; p < 0.0001; Fig. 8C), consistent with this population being subject to negative selection. Although proportions of the DN and CD4^+^ populations of thymic iNKT cell numbers were significantly increased in α-GalCer treated mice (p < 0.0001), they showed modest reductions in terms of absolute numbers (CD4^+^ NS; DN p < 0.01). In contrast, numbers of iNKT cells in the periphery were greater in NOD.*Va14*^*tg*^ mice injected with α-GalCer, increasing from 1.85 ± 0.16 × 10^6^ in livers of the controls to 3.50 ± 0.28 × 10^6^ in those of the treated mice (p < 0.005) and from 4.65 ± 0.48 × 10^6^ to 7.24 ± 0.53 × 10^6^ in the spleens (p < 0.005), consistent with antigen-induced activation and proliferation (Fig. 8C).

In a separate experiment, the effects of i.v. injection of α-GalCer were examined in NOD.*Va14*^*tg*^ mice bearing a targeted gene deficiency of CD1d (NOD.*Va14*^*tg*^.*Cd1d*^−*/*−^ mice). No significant changes in iNKT cell numbers were observed in either the thymi or the periphery, consistent with the changes observed in NOD.*Va14*^*tg*^ mice being dependent on α-GalCer presentation by CD1d (Supplementary Fig. 1).

Peripheral activation of large numbers of T cells by systemic administration of a polyclonal activator can cause bystander thymocyte death by eliciting a “cytokine storm”^80^. In order to minimise the effects of systemic T cell activation in our model of enhanced negative selection of immature iNKT cells, NOD.*Va14*^*tg*^ mice were subjected to intrathymic injection of α-GalCer and flow cytometric analysis of numbers and subsets of iNKT cells 40 h later (Fig. 8D). The effects of intrathymic injection of α-GalCer on thymic iNKT cells were similar to those of systemic α-GalCer administration. In terms of absolute numbers, thymic iNKT cell numbers fell from 3.97 ± 0.70 × 10^8^ (mean ± SEM) to 2.31 ± 0.36 × 10^8^ cells. The proportion of iNKT cells that were DP fell from 58.7 ± 2.5 to 18.5 ± 1.2% (p < 0.005; Mann–Whitney U Test; n = 5–7). Again, the proportions of CD4 SP and DN thymic iNKT cells increased, from 13.7 ± 0.6 to 29.5 ± 0.7% (p < 0.005) and from 23.3 ± 1.6 to 47.1 ± 0.7% (p < 0.005) respectively. The specificity of the deletion induced by intrathymic α-GalCer on developing iNKT cells, as distinct from conventional T cells, was illustrated by the relative depletion of DP^hi^CD24^hi^CD44^lo^ NKT cells (> 82%) compared to DP^hi^CD24^hi^CD44^lo^ conventional T cells (< 19%).

### Transition 3: Differentiation

The expression levels of all 35,556 transcripts across Transition 3 (immature CD4 + NKT cells to DN NKT cells) were compared pairwise by Mann–Whitney U test, and those with a U statistic of zero (i.e. no overlap between groups) were shortlisted for ranking by Student t Test. Across this transition, 6,904 transcripts generated a Mann–Whitney U score of zero, and of these, 1,849 were highly differentially expressed (HDE; p < 1.0 × 10^–6^, by t-Test). Of the HDE transcripts, 1,270 were up-regulated in DN NKT cells compared to CD4^+^ NKT cells, while 579 transcripts were down-regulated. Only 47 of these HDE genes were in the network, 27 in the “Pink” module and 15 in the “Purple” module. We therefore submitted all (both up and down-regulated) genes lists to DAVID Bioinformatics Resources v6.7^49^ for gene ontology analysis of functional annotation clustering.

As a generalisation, the gene ontology analysis of functional annotation clustering of the downregulated genes was unremarkable. In no case was the Bonferroni corrected Enrichment p value less than 0.001. This unexceptional finding was re-iterated when comparing our gene list with that of Cohen et al.^62^, Stage 1 genes. While we could confirm 186 of their 202 genes (Supplementary Table 1), as significantly downregulated by Stage 1, our data indicated that most were down-regulated between the DPNKT and CD4NKT stage, (6 were downregulated in Transition 1 alone, and 7 showed down-regulation but did not meet our criteria for stringent cut-off, in both Transition 1 and Transition 2). None of the genes mentioned in their gene list, however, were down-regulated between CD4 and DN stage in our dataset.

In stark contrast to this, the list of upregulated genes was dominated by those required for the production of ribosomes: The top-ranked Annotation Cluster (Enrichment Score 29.06) contained the GO term Nucleolus (GO:0005730; enrichment 5.86-fold; p < 1.54 × 10^–34^ Bonferroni corrected) as well as its various parent terms (nuclear lumen, intracellular non-membrane bound organelle, intracellular organelle lumen). Similarly, the second top-ranked Annotation Cluster (Enrichment score 23.16) contained the GO term rRNA Processing (GO:0006364; enrichment 10.11-fold; p < 2.53 × 10^–19^), as well as its various parental terms (ncRNA Processing, rRNA Metabolic Process, RNA Processing, Ribonucleoprotein Complex Biogenesis, Ribosome Biogenesis and ncRNA Metabolic Process). In a comparison with Cohen’s upregulated Stage 1 gene set^62^, we confirmed 123/146 transcripts as significantly upregulated in our dataset (Supplementary Table 2; while most genes showed upregulation in Transition 2, 9 were significantly highly differentially upregulated in Transition 1, and although most remained unchanged in Transition 3, 18 of the transcripts were again highly significantly up-regulated during this transition. These included *Plac8*, *Enpp1*, *St6galnac4*, *Slc22a3*, *Dhrs11*, *Agpat2*, *Sccpdh*, *Maf*, *Trf*, *Top1mt*, *Samsn1*, *Tgm3*, *Ptpn13*, *Zbtb10*, *Fcrl1*, *Dnmt3b*, *Ehd4*, *Ntrk3*, genes associated with Metabolic Pathways, Calcium Ion Binding and Positive Regulation of Gene Expression. Nine transcripts identified as upregulated by Stage 1 in Cohen’s dataset^62^, that were also up-regulated by our CD4NKT cells (*Gpr114*, *Itgae*, *Ar*, *Appl2*, *St8sia6*, *Dse*, *App*, *Acsbg1*, *Adam19*; genes associated with the terms Glycoprotein, Signal, Disulphite bond, Membrane, Transmembrane and Protein binding), were again significantly downregulated when transitioning to DN NKT cells.

Although these data are consistent with functional differentiation across this transition, there is no evidence of major differences in the amount of proteins being produced by the CD4 + and DN subsets of mature iNKT cell after export^52^. An alternative possibility is that the production of ribosomes reflects the maturity of the cells, and the DN population, being derived from the CD4 NKT cells is therefore likely to be, on average, more mature.

### iNKT1, iNKT2, iNKT17 branch point

Most of the iNKT1 cells resemble Stage 3 cells (NK1.1^+^ and CD44^hi^), while iNKT2 and iNKT17 cells are both NK1.1^−^ and therefore resemble those of Stage 1 or Stage 2 NKT cells and can be mistaken for them^4^. There is however, some controversy surrounding the developmental pathway of these subsets. Certain data substantiates a developmental pathway theory^81–84^ while other researchers favour a lineage differentiation pathway instead^4–6,85–91^, with questions remaining as to the branch point at which iNKT1, iNKT2 and iNKT17 cells emerge. iNKT1 cells are defined as *NK1.1*^+^*Tbet*^+^^4,7,83^ with upregulated *Erg2*^81^, *FcεR1γ*^7^ and the micro-RNA *Let-7*^92^, and express the cytokines, IFNγ^4,7,83^ and CCL5^7,83^, iNKT2 cells are NK1.1^−^ with upregulation of either *Gata3*^4^*, Irf-4*^4,83^ or *Zbtb16*^7,83^ and expression of IL4^4,7,83^ and IL13^7,83^; while iNKT17 cells are defined as *NK1.1*^−^* Rorγt*^+^^4,7,83,85^ with upregulated *IL23R*^7,83^, *SerpinB1*^7,83^ and *Bcl11b*^88^, and they express IL17^7,83^. We used these genes as identifiers as possible indicator as to branch point for the individual subsets (Supplementary Fig. 2). Expression levels remained unchanged for most identifiers across these transitions. *Tbx21* was upregulated in Transition 1 and remained consistently so throughout, while *Gata3* showed high level expression in all subsets, with particular upregulation in Transition 3, confirming Cameron and Godfey’s previous observations^93^. Other identifiers were upregulated, but showed little difference between our early CD4NKT and DNNKT populations; *Rorc* was most highly expressed in Population 1, downregulating in Transition 2 and again in Transition 3; and *Bcl11b* was most highly expressed in DP T cells, with slight down-regulation in Transition 1 and then remaining constant until Transition 3 when it down-regulated again, although still remaining highly expressed. All other genes were of low to medium expression, showing no difference between subsets.

In summary, these observations may be an indication that there is no subset differentiation this early in the developmental pathway, although our data is unable to provide support for either of the two theories.

## Discussion

This manuscript examines the transcriptional profiles of immature thymic iNKT cell subsets in a novel Vα14 TCR transgenic strain, and reveals functional characteristics initiated by positive selection on CD4^+^CD8^+^ (double positive, DP) thymocytes that are distinct from conventional T cells. Previously published data suggesting that the thymic DP^hi^CD24^hi^CD44^lo^NK1.1^−^CD1d-tet^+^TCRβ^+^ population represents iNKT cells that have not yet undergone thymic selection can be summarised as follows:

1. The population lacks expression of the developmental markers typical of mature iNKT cells, viz: NK1.1 and CD44.
2. It expresses the CD24 marker, which is characteristic of conventional T cells prior to selection.
3. Quantitative PCR analysis of Vα14-Jα18 encoded transcripts of sorted DP^hi^ thymocytes found equivalent proportions in WT and *Cd1d*^−*/*−^ mice^28^. Similarly, multiple TCR rearrangements could be detected in DP^hi^ thymocytes of WT mice, in contrast with only a single Vα14-Jα18 rearrangement in the DP^dull^ population of WT mice^28^.

The combination of transgenic expression of an NKT-associated Vα14-Jα18 TCRα chain cDNA on the SLAM-deficient NOD strain background^23,24,38^ resulted in greatly increased numbers of immature DP iNKT cells. The behaviour of these cells under experimental conditions was consistent with them not yet having undergone selection, in as much as:

1. Cells of this phenotype were not found outside the thymus, despite very large numbers of them being found within it;
2. Their numbers were more than doubled by targeted deletion of CD1d, which is the antigen presentation glycoprotein required for iNKT cell positive selection;
3. They were depleted to about half their numbers by systemic or intrathymic injection of the strong iNKT cell glycolipid antigen α-GalCer in a CD1d-dependent manner; and
4. They showed only three of 44 transcriptional hallmarks of selection.

NOD.*Va14*^*tg*^ mice provided a model of iNKT cell selection in which sufficient pre-selection cells were available that the stages and processes involved could be clearly distinguished. Evidence supporting this use of the model comes from the relatively large number of transcripts differentially expressed between thymic TCRβ^+^DP^hi^CD24^hi^ populations that either did, or did not, bind CD1d-tet. This comparison provided evidence of successful TCR signalling in thymic DP^hi^CD24^hi^NK1.1^−^ NKT cells, including TCRα allelic exclusion and *Rag1* down-regulation.

iNKT cells are positively selected by ligating CD1d expressed on DP cortical thymocytes, which account for > 80% of all thymocytes. The accumulation of pre-selection DP iNKT cells in NOD.*Va14*^*tg*^ mice is consistent with an additional rate-limiting step or signal. One possible candidate is the SLAM-SLAM homotypic interaction between immature iNKT cells and selecting DP thymocytes. Alternatively, other co-stimulators or cytokines, such as IL7 or IL15, may be required.

Although others^62^ have identified changes in gene expression between DP T and CD24^lo^CD44^lo^ Nk1.1^−^ cells (Stage 1), the presence of greatly expanded numbers of very immature iNKT cells in NOD.*Va14*^*tg*^ mice provided an opportunity to study the very earliest identifiable stages, prior to Stage 1, of iNKT cell commitment and differentiation. Thus, while we were able to substantiate other investigators' findings, we could also determine the interval of the occurrence, and in addition, determine the most significant functional changes at each transition. In this way, we could help dissect factors associated with, and contributing to the numbers and function of this important immunoregulatory population.

Transcriptional analysis comparing immature DP T cells and immature DP iNKT cells revealed a reduction in expression of the electron transport genes, such as NADH Dehydrogenase (*Nd1*, *Nd2*, *Nd4l*, *Nd5*), Cytochrome C Oxidase (*Cox1*, *Cox2*, *Cox3*) and ATP Synthase 6 (*Atp6*) suggesting reduced mitochondrial oxidative phosphorylation. This is a characteristic of the Warburg phenomenon, in which cancerous or other rapidly proliferating cells increase aerobic glycolysis at the expense of oxidative phosphorylation^94,95^. In addition, the most strongly differentially expressed gene identified across Transition 1 encoded the transcription factor E2F1, which was highly significantly upregulated, lending support for increased proliferation in DP iNKT cells. E2F1, E2F2 and E2F3 act in a functionally redundant manner to enhance the expression of many genes required for G_1_ to S phase cell cycle progression, proliferation and development, including *Cdc6*, *Ccne1* and *Myb*^56,96^. The growth regulatory function of the Rb tumour suppressor protein is mediated by its binding to E2F transcription factors, so that while overexpression of E2F1, unopposed by increased RB expression, results in increased proliferation, so does *E2f1* gene deletion^97,98^. Consistent with increased *E2f1* expression, E2F1- regulated genes, such as *Cdc6* and *Ccne1* were also significantly upregulated, as were genes encoding proteins critical for spindle formation and chromosome segregation (such as *Cenpm)* and DNA replication (such as *Rpa2*).

The role of the E2F pathway in mediating T cell proliferation has been studied in *E3f1/E2f2* double knockout mice. It appears to play a role in homeostatic proliferation but not in proliferative responses to exogenous antigen, as homeostatic proliferation in *E3f1/E2f2* double knockout mice is severely reduced^99^, but proliferation in response to exogenous antigen is not^99,100^. While homeostatic proliferation of naive T cells requires both IL7 signalling^101^ and TCR stimulation by MHC-self peptide^102^, homeostatic proliferation of memory CD4 T cells is dependent on TCR stimulation by MHC-self peptide alone^103^. As homeostatic proliferation of all T cells, including memory CD4 T cells is impaired in *E3f1/E2f2* double knockout mice, the E2F pathway must play a role in mediating T cell proliferation in the context of homeostatic proliferation^98^. The early processes of T cell selection resemble those of homeostatic proliferation, in that they are mediated by MHC-self peptide recognition and stimulate proliferation. Activation of the E2F pathway in Transition 1 of iNKT cells suggests that T cell signalling had occurred.

While Benlagha et al.^34^ previously reported that DP^lo^CD24^hi^NK1.1^−^ NKT cells in the thymi of B6 newborn mice were non-dividing, our functional validation by in vivo BrdU incorporation, confirmed our hypothesis that the immature thymic DP^hi^ iNKT cells in NOD.*V*α*14*Tg mice have increased proliferation. Transcriptional evidence of allelic exclusion of competing TCR Vα chains in immature DP iNKT cells was provided by the widespread down-regulation of non-NKT cell TCR *V*α genes in Transition 1. This finding was confirmed with flow cytometry by the almost complete absence of immature DP NKT cells and mature TCR Vα14-Jα18-expressing NKT cells co-expressing other TCRα chains, such as TCR Vα2, Vα3.2 and Vα8.3 (data not shown). Down-regulation of genes related to the electron transport chain, the activation of the E2F pathway, T cell signaling and the down-regulation of non-NKT associated TCR Vα genes suggests that successful TCR signaling occurs across Transition 1.

Transcriptional analysis suggests that positive selection and lineage commitment of iNKT cells occurs during the transition from immature DP NKT cells to immature CD4 NKT cells. Gene Ontogeny analysis of up-regulated HDE genes across this transition identified a wide range of functional lymphocyte-associated membrane proteins, such as Toll-like receptors, cytokine receptors, chemokine receptors, integrins and leukocyte differentiation markers. In addition, there was a predominance of genes associated with the immunomodulatory and innate-like properties of iNKT cells, such as *Tlr1, Nkg7, Sema4a, Art2b, S1pr1* and *Zbtb16*. Commitment to the iNKT cell lineage is associated with the expression of the transcription factor, PLZF, encoded by *Zbtb16*^61^. While there was no significant difference in expression of *Zbtb16* between immature DP T cells and immature DP NKT cells, *Zbtb16* was up-regulated more than 13-fold during the subsequent transition from immature DP NKT cells to the immature CD4 NKT stage.

The coordinated expression of the innate-like lymphocyte-associated transcription factor PLZF and the subsequent up-regulation of a wide range of cell-surface functional receptors associated with iNKT cell immunobiology combine to provide evidence that iNKT cell lineage commitment occurred across Transition 2, between DP NKT and immature CD4 NKT cell stage. The occurrence of lineage commitment at Transition 2 raised the issue of the timing of the NKT cell selection event. Many of the up-regulated genes expressed by the immature CD4 NKT cells have been previously reported as associated with the positive selection of conventional T cells^64,104^ consistent with iNKT cell selection also occurring across Transition 2.

Positive and negative selection of iNKT cells has been previously studied. iNKT development is markedly impaired by the absence of the antigen presenting molecule CD1d^25,27,28,43,105–107^ or by the early administration of α-GalCer^79,108^. In studies of WT mice, the very low numbers of DP iNKT cells prohibited examination of the effect of targeted deletion of CD1d and administration of α-GalCer on DP iNKT cells^79^.

In the absence of CD1d, immature DP iNKT cells in NOD.*Va14*^*tg*^*.Cd1d*^−/−^ mice did not progress to negative selection or maturation and export, resulting in accumulation in the thymus. This may be due to premature transgenic TCRα expression mimicking the pre-TCRα chain^67^ and altering the frequencies of TCRβ chain usage^68^. This, however, will require further investigation. The almost complete absence of peripheral iNKT cells in NOD.*Va14*^*tg*^*.Cd1d*^−/−^ mice is consistent with failed positive selection of this population in the absence of CD1d. Together, these data are consistent with the hypothesis that positive selection of iNKT cells occurs between the immature DP and the CD4 single positive stages.

In summary, our transcriptional regulatory network approach of iNKT cell development mapped TCR signal modulation or “tuning” to the transition from DP T to DP NKT cells, while positive selection and lineage commitment were associated with the transition from DP NKT to CD4 NKT cells. We speculate that this early signalling event in NKT cells may constitute “validation” of an effective TCR prior to functional differentiation, and that a similar process might also occur in conventional T cells.

## Supplementary Information

Supplementary Information.

## Data Availability

The data discussed in this publication have been deposited in NCBI's Gene Expression Omnibus (Dinh et al., 2021) and are accessible through GEO Series accession number GSE106720 (https://www.ncbi.nlm.nih.gov/geo/query/acc.cgi?acc=GSE106720).
