# Supplementary material for: Modulation of TCR signalling components occurs prior to positive selection and lineage commitment in iNKT cells
Source: Sci Rep. 2021 Dec 8;11:23650. doi: 10.1038/s41598-021-02885-w (PMC8655039; doi:10.1038/s41598-021-02885-w)
Supplement: Supplementary file 1 — Supplementary Information. [file 41598_2021_2885_MOESM1_ESM.pdf]

Modulation of TCR signalling components occurs prior to positive selection and lineage commitment in iNKT cells

Xuyen T Dinh[1,2], Dragana Stanley[3], Letitia D Smith[1], Morgane Moreau[1], Stuart P Berzins[4,5], Adrian Gemiarto[1], Alan G Baxter [1,*], and Margaret A Jordan[1,*,**]

1. Molecular & Cell Biology, College of Public Health, Medical & Veterinary Sciences, The Science Place, building 142, James Cook University, Townsville, QLD 4811, Australia.
2. Hai Duong Medical Technical University, Hai Duong, Viet Nam

3. Central Queensland University, School of Medical and Applied Sciences, Rockhampton, Queensland, 4702, Australia.
4. School of Science, Psychology and Sport, Federation University Australia, Ballarat, VIC 3350, Australia.
5. Peter Doherty Institute for Immunity and Infection, University of Melbourne, Parkville, VIC 3050 Australia.

* These authors contributed equally
** Correspondence to MAJ:

Phone: 61-7-4781 5965
Fax: 61-7-4781 6078
Email: Margaret.Jordan@jcu.edu.au

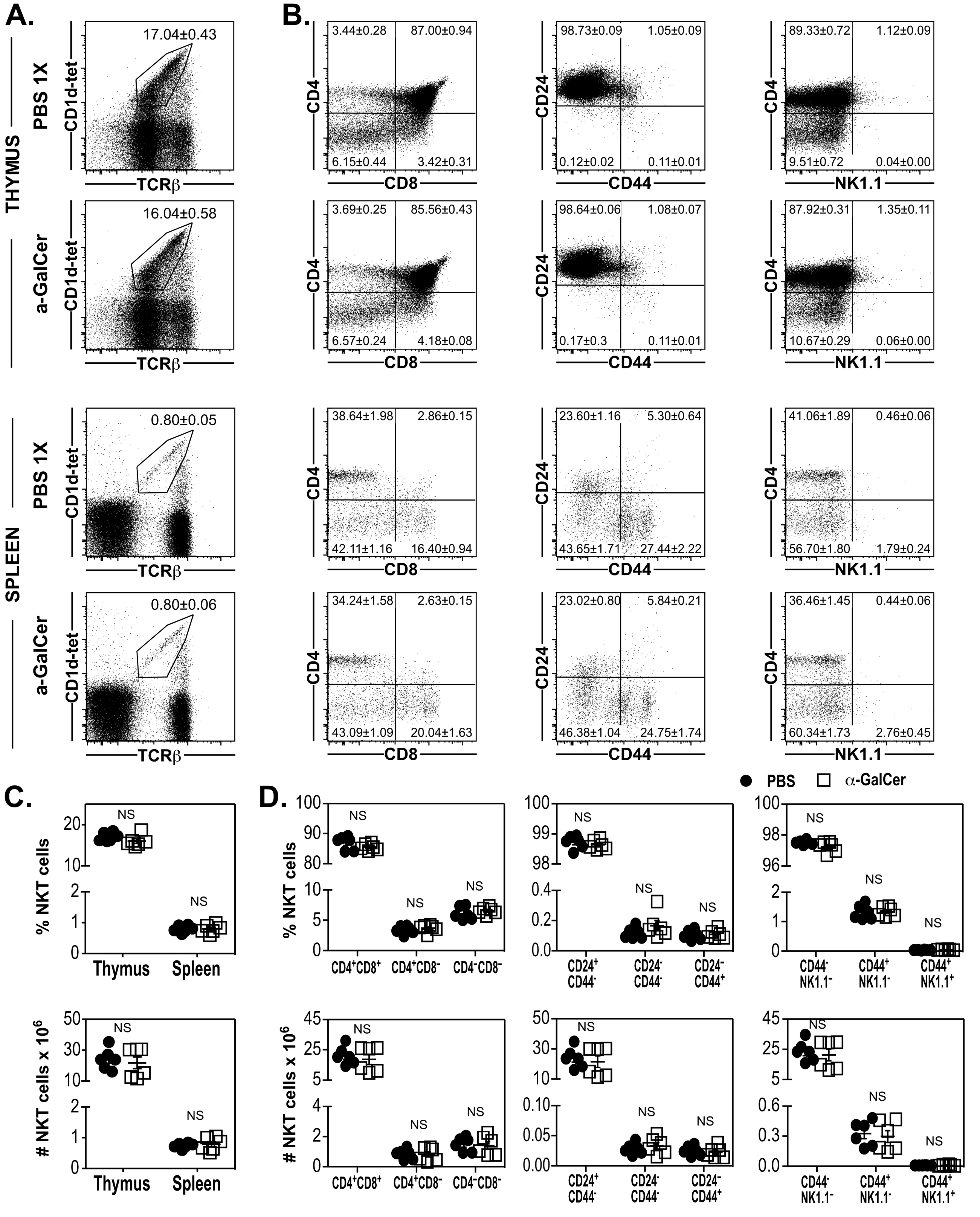

Supplementary Figure 1, Dinh et al.

SUPPLEMENTARY FIGURE 1:

Effect of i.v. α-GalCer administration on NKT cells in NOD. *Va14^{tg}.Cd1d$^{-/-}$* mice. A. Representative FACS plots show the mean frequency (±SEM) of thymic and splenic NKT cells in total cells from the thymi and spleens, respectively, of PBS and α-GalCer injected NOD.*Va14^{tg}.Cd1d$^{-/-}$* mice. **B**. Representative FACS plots show the mean frequency (±SEM) of thymic and splenic NKT cell subsets in total NKT cells from the thymi and spleens, respectively, of PBS and α-GalCer injected NOD.*Va14^{tg}.Cd1d$^{-/-}$* mice. **C**. Dot plots show mean frequency and absolute numbers of thymic and splenic NKT cells. **D**. Dot plots show mean frequency and absolute numbers of thymic and splenic NKT cell subsets. (n=6 mice/group)

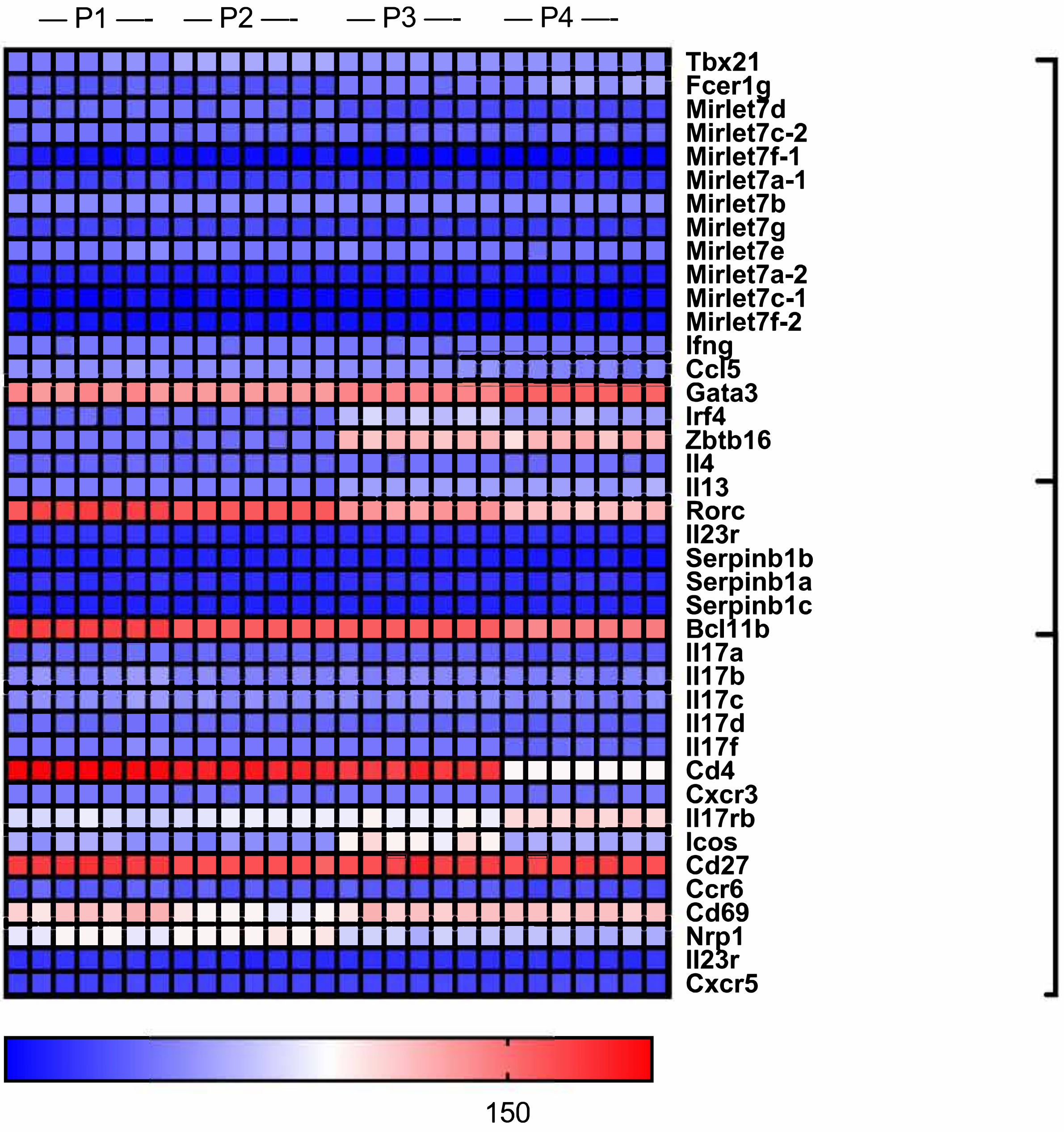

Supplementary Figure 2, Dinh et al

SUPPLEMENTARY FIGURE 2:

Heat map showing gene expression profiles of the four different cell types across the three transitions for the gene identifiers of NKT subsets. NKT1 as distinguished by *Tbx21*$^{+}$*Erg*$^{+++}$ *Fcer*g^{+++}*mirLet7*$^{+++}$*Ifng*$^{+}$*Ccl5*$^{+}$; NKT 2 as distinguished by (*Gata3*$^{+++}$or *Irf4*$^{+++}$ or *Zbtb16*$^{+++}$)*IL4*$^{+}$*IL13*$^{+}$ and NKT17 by *Rorc*$^{+}$*IL23R*$^{+++}$*SerpinB*$^{+++}$*BclIIB*$^{+++}$*IL17*$^{+}$ (7 samples/group). (Figure generated using Prism_GraphPad V9.2.0 (283) July 15, 2021; https://www.graphpad.com/scientific-software/prism/).

Supplementary Table 1: Genes from our dataset in common with Cohen's downregulated genes by Stage 1 (Cohen et al, 2012)

				Regulation in T1			Regulation in T2			Regulation in T3				Cohen et al 2012: Genes	
probeset_id	seqname	start	stop	p-value(DPT vs. DPNKT)	FC to DPNKT	Direction in DPNKT	p-value(DPNKT vs. CD4NKT)	FC to CD4NKT	Direction in CD4NKT	p-value(CD4NKT vs. DNNKT)	FC to DNNKT	Direction in DNNKT	Gene Symbol	FC (Stage 1 vs DPT)	p-value
10511901	chr4	32804035	32950826	5.25E-17	-2.67496	DOWN	1.29E-10	-1.7472	DOWN	0.271702	1.07033	UP	Ankrd6	-2.82	0.01874
10546510	chr6	94604529	94700145	7.18E-17	-2.0333	DOWN	1.95E-10	-1.49131	DOWN	6.31E-14	-2.07996	DOWN	Lrig1	-2.17	0.0057
10581455	chr8	106130402	106136974	3.66E-16	-2.73131	DOWN	2.49E-08	-1.59438	DOWN	0.0794583	-1.13216	DOWN	Esrp2	-3.01	0.02515
10557326	chr7	125552282	125579474	5.45E-16	-2.12172	DOWN	5.82E-07	-1.34049	DOWN	4.28E-12	-1.99956	DOWN	Il4ra	-3.97	0.0016
10439239	chr16	35694903	35769356	8.58E-16	-2.21403	DOWN	3.91E-11	-1.72987	DOWN	4.79E-11	-1.9363	DOWN	Dirc2	-3.83	0.00633
10506058	chr4	98395826	98719603	1.38E-15	-2.26438	DOWN	0.0262496	-1.12117	DOWN	3.14E-06	-1.43624	DOWN	Inadl	-2.32	0.00411
10414796	chr14	53032902	53033418	1.64E-15	-1.94026	DOWN	0.147093	-1.06077	DOWN	9.05E-05	-1.25673	DOWN	EG547328	-2.49	0.00053
10352661	chr1	189728268	189876695	5.36E-15	-1.87272	DOWN	3.42E-12	1.70772	UP	7.48E-07	-1.38662	DOWN	Ptpn14	-2.26	0.019
10396936	chr12	81026828	81186410	5.55E-15	-4.86905	DOWN	2.83E-10	-2.93084	DOWN	0.0943125	-1.23586	DOWN	Smoc1	-5.38	0.01643
10553015	chr7	45570358	45589712	6.27E-15	-1.74565	DOWN	0.0204404	-1.09189	DOWN	0.199091	1.05841	UP	Bcat2	-2.02	0.00014
10513692	chr4	63414910	63495951	2.12E-14	-1.60201	DOWN	2.04E-10	-1.41561	DOWN	3.94E-06	-1.26405	DOWN	Whrn	-2.78	0.0017
10514713	chr4	103038065	103114266	3.12E-14	-2.37833	DOWN	1.58E-12	-2.30904	DOWN	5.54E-05	-1.43169	DOWN	Wdr78	-11.36	0.01079
10400762	chr12	69803750	69893163	8.50E-14	-2.69547	DOWN	5.84E-13	-2.87815	DOWN	2.81E-09	-2.28574	DOWN	Map4k5	-6.99	0.00018
10577226	chr8	13952009	13974690	8.96E-14	-2.81281	DOWN	1.65E-09	2.07647	UP	1.81E-09	-2.42482	DOWN	2610019F03Rik	-2.53	0.00523
10366712	chr10	122678805	122942539	1.14E-13	-1.64771	DOWN	0.277775	1.04124	UP	1.13E-08	-1.47735	DOWN	Ppm1h	-3.37	0.00337
10390535	chr11	97989578	97996173	1.21E-13	-2.21878	DOWN	1.91E-06	-1.45116	DOWN	1.62E-11	-2.43618	DOWN	Arl5c	-5.92	0.00048
10486595	chr2	120732816	120850604	3.28E-13	-1.58738	DOWN	3.78E-09	-1.39422	DOWN	0.0985789	-1.07726	DOWN	Ttbk2	-2.47	0.009
10589099	chr9	108783796	108806337	3.85E-13	-1.46688	DOWN	0.00065459	-1.12454	DOWN	0.208436	-1.04784	DOWN	Ip6k2	-2.09	0.00467
10382532	chr11	115462474	115474154	4.25E-13	-1.97456	DOWN	1.34E-08	-1.58567	DOWN	3.02E-05	-1.40009	DOWN	Slc16a5	-13.51	0.00008
10404132	chr13	24327404	24471013	4.97E-13	-1.53488	DOWN	3.28E-07	-1.27268	DOWN	1.95E-11	-1.66276	DOWN	Cmah	-2.34	0.0092
10348321	chr1	87853301	87944490	7.86E-13	-1.55169	DOWN	0.00764354	-1.10874	DOWN	9.06E-06	-1.27854	DOWN	Dgkd	-2.27	0.00338
10472097	chr2	52858143	53133803	1.06E-12	-2.30287	DOWN	1.35E-11	-2.36207	DOWN	0.0585944	-1.17902	DOWN	Fmnl2	-3.32	0.00288
10575598	chr8	111536842	111624294	1.07E-12	-1.55807	DOWN	0.00145221	-1.14011	DOWN	3.72E-08	-1.43507	DOWN	Znrf1	-2.88	0.00033
10445078	chr17	37045966	37074305	1.28E-12	-2.00101	DOWN	0.0160322	1.15993	UP	5.79E-09	-1.88692	DOWN	Gabbr1	-2.07	0.00199
10426611	chr15	98632220	98644530	2.19E-12	-1.85584	DOWN	1.01E-10	-1.81382	DOWN	1.48E-10	-2.03556	DOWN	Cacnb3	-2.05	0.03238
10399897	chr12	31926460	31950223	2.56E-12	-1.86825	DOWN	0.0169097	1.14701	UP	3.35E-05	-1.3986	DOWN	Hbp1	-2.09	0.01281
10368199	chr10	21124930	21160984	2.87E-12	-1.31253	DOWN	6.06E-13	-1.41168	DOWN	1.06E-05	1.17399	UP	Myb	-2.51	0.00044
10425410	chr15	80572594	80650298	3.58E-12	-1.32948	DOWN	0.00284898	1.08607	UP	5.58E-07	-1.23169	DOWN	Grap2	-2.25	0.00005
10497920	chr3	38449261	38484801	4.11E-12	-1.61437	DOWN	0.0194732	1.11069	UP	7.75E-05	-1.2785	DOWN	Ankrd50	-3.57	0.00048
10435094	chr16	32644643	32683493	6.37E-12	-1.77072	DOWN	0.383891	1.04618	UP	0.0593459	1.15523	UP	Tnk2	-2.12	0.01991
10373542	chr10	128720134	128744056	7.14E-12	-1.54132	DOWN	2.51E-08	1.38512	UP	4.31E-07	-1.3957	DOWN	Dgka	-2.02	0.00105
10390519	chr11	97923238	97986380	8.04E-12	-1.86999	DOWN	3.56E-10	-1.8256	DOWN	1.21E-08	-1.82709	DOWN	Plxdc1	-3.6	0.00019
10521481	chr5	37050916	37125295	8.39E-12	-1.42224	DOWN	0.00835148	-1.09641	DOWN	0.00248929	-1.14126	DOWN	Jakmip1	-2.07	0.0012
10479010	chr2	172979842	172993571	1.33E-11	-2.74596	DOWN	2.56E-09	-2.44677	DOWN	6.36E-07	-2.18938	DOWN	Spo11	-16.95	0.00057
10488892	chr2	155634277	155692360	1.58E-11	-1.3984	DOWN	0.00011405	-1.15751	DOWN	0.00155795	-1.14726	DOWN	Trpc4ap	-2.65	0.00658
10529375	chr5	34175964	34187710	1.67E-11	-1.76893	DOWN	0.0361694	1.12627	UP	2.03E-05	-1.41956	DOWN	Mxd4	-2.25	0.00351
10438639	chr16	22468446	22657221	2.04E-11	-1.46191	DOWN	1.31E-16	-2.22706	DOWN	5.31E-08	-1.4215	DOWN	Dgkg	7.35	0
10486112	chr2	118528757	118549678	2.22E-11	-3.39869	DOWN	0.0615883	-1.25666	DOWN	0.0413391	-1.35833	DOWN	Bmf	-3.32	0.01243
10510552	chr4	150406340	150619489	2.73E-11	-1.54345	DOWN	2.02E-06	1.30392	UP	7.29E-05	-1.27965	DOWN	Rere	-2.7	0.0159
10503259	chr4	11156441	11174379	3.05E-11	-1.48499	DOWN	0.00046256	-1.1695	DOWN	2.32E-05	-1.28145	DOWN	Trp53inp1	-3.41	0.00047
10469457	chr2	16356304	16752305	3.10E-11	-1.43608	DOWN	3.14E-07	-1.28639	DOWN	4.38E-06	-1.29328	DOWN	Plxdc2	-7.41	0.004
10378053	chr11	72042502	72047371	3.24E-11	2.99192	DOWN	9.08E-09	-2.57598	DOWN	0.0317027	-1.34475	DOWN	Fam64a	-2.27	0.02867
10408693	chr13	36867187	37049204	4.83E-11	-2.15489	DOWN	8.92E-10	-2.16098	DOWN	5.67E-08	-2.09715	DOWN	F13a1	-20	0.00249
10345442	chr1	36068400	36106446	6.72E-11	-1.89279	DOWN	0.00112775	-1.27104	DOWN	0.033028	-1.19437	DOWN	Hs6st1	-2.8	0.03408
10585778	chr9	57940135	57962865	7.13E-11	-1.65546	DOWN	2.20E-13	-2.2092	DOWN	0.0504762	1.13661	UP	Sema7a	-6.94	0.00012
10362829	chr10	42678917	42702459	8.02E-11	-1.33887	DOWN	1.28E-06	-1.21494	DOWN	0.00327108	-1.12627	DOWN	Ostm1	-2.28	0.01718
10461071	chr19	7420534	7425904	8.38E-11	-1.35792	DOWN	1.72E-08	-1.30673	DOWN	0.513568	1.02547	UP	2700081O15Rik	-2.54	0.00648
10380514	chr11	95337018	95381872	8.40E-11	-1.45885	DOWN	0.65309	-1.01764	DOWN	0.0178444	-1.12719	DOWN	Fam117a	-2.92	0.00181
10358928	chr1	154395952	154726356	9.09E-11	-2.5054	DOWN	0.437277	-1.07697	DOWN	0.305603	-1.12725	DOWN	Cacna1e	-3.29	0.00012
10423293	chr15	25622926	25813671	9.94E-11	-1.57289	DOWN	0.00066583	-1.20184	DOWN	0.00750975	-1.18124	DOWN	Myo10	-2.17	0.02112
10541877	chr6	125215581	125240544	1.42E-10	-2.3693	DOWN	0.812722	-1.02184	DOWN	0.00276469	-1.44773	DOWN	Vamp1	-5.78	0.00051
10523647	chr5	103754329	103851123	1.52E-10	-1.32868	DOWN	2.17E-09	-1.33445	DOWN	1.56E-10	-1.49999	DOWN	Aff1	-3.25	0.00159
10514221	chr4	86656565	86670059	1.59E-10	-1.47463	DOWN	1.02E-12	-1.80494	DOWN	0.0525894	-1.05676	DOWN	Plin2	-2.33	0.01514
10390186	chr11	95830072	95842160	1.86E-10	-1.45977	DOWN	1.60E-05	-1.24649	DOWN	1.29E-06	-1.37915	DOWN	Abi3	-3.3	0.00101
10480347	chr2	18842260	18998126	2.23E-10	-1.27661	DOWN	1.50E-09	-1.2946	DOWN	6.60E-05	-1.16913	DOWN	Pip4k2a	-2.04	0.00191
10586616	chr9	67840412	67995389	2.25E-10	-1.73832	DOWN	0.0578183	-1.12579	DOWN	0.0118271	-1.21894	DOWN	Vps13c	-2.15	0.0082
10438738	chr16	23965052	23988633	2.31E-10	-1.66485	DOWN	8.64E-11	-1.87708	DOWN	1.00E-05	-1.4611	DOWN	Bcl6	-9.17	0
10510129	chr4	144892827	144928209	2.47E-10	-1.58629	DOWN	6.29E-13	-2.08679	DOWN	1.00E-12	-2.40069	DOWN	Dhrs3	-4.5	0.00262
10547100	chr6	115954811	115994964	2.88E-10	-1.71563	DOWN	1.56E-11	-2.09063	DOWN	8.72E-13	-2.82975	DOWN	Plxnd1	-15.38	0.00096
10383970	chr11	5191552	5261610	2.97E-10	-1.40509	DOWN	3.65E-10	-1.48356	DOWN	4.66E-08	-1.44112	DOWN	Kremen1	-6.58	0.00246
10437160	chr16	95702486	95721049	3.26E-10	-1.38733	DOWN	6.49E-13	-1.69711	DOWN	5.22E-12	-1.78666	DOWN	Ets2	-6.99	0.00022
10574825	chr8	105686274	105698165	4.02E-10	-1.42103	DOWN	0.00086809	-1.16118	DOWN	5.07E-07	-1.39219	DOWN	Rltpr	-2.53	0.00021
10486061	chr2	117279990	117342877	4.31E-10	-1.30727	DOWN	0.0453116	1.06507	UP	0.753157	-1.01159	DOWN	Rasgrp1	-3.41	0.00055
10521440	chr5	35893319	36003922	4.50E-10	-1.44927	DOWN	0.00058683	-1.17986	DOWN	0.216652	-1.066	DOWN	Afap1	-2.72	0.0023
10364502	chr10	79793588	79820912	4.67E-10	1.34771	DOWN	4.42E-13	-1.65045	DOWN	1.17E-05	1.25592	UP	Palm	-2.7	0.00012
10355141	chr1	64035661	64121418	4.74E-10	-1.60927	DOWN	1.98E-08	-1.57233	DOWN	4.39E-07	-1.57817	DOWN	Klf7	-3.82	0.00783
10571321	chr8	35375741	35388145	4.98E-10	-1.78427	DOWN	0.0266892	-1.1661	DOWN	0.016188	-1.22792	DOWN	Ppp1r3b	-3.48	0.00109
10568668	chr7	133883199	134225097	6.41E-10	-1.83275	DOWN	3.20E-12	-2.55252	DOWN	0.0018954	-1.34306	DOWN	Adam12	-2.36	0.01233
10593988	chr9	57750709	57765860	6.56E-10	-1.39128	DOWN	5.83E-11	-1.55351	DOWN	1.70E-07	-1.40795	DOWN	Clk3	-3.61	0.00024
10426704	chr15	99224952	99242824	7.49E-10	-1.6506	DOWN	0.57668	-1.03297	DOWN	1.38E-06	-1.57891	DOWN	Kcnh3	-2.24	0.00158
10454741	chr18	34777052	34838989	8.44E-10	-1.38066	DOWN	0.00401225	-1.12654	DOWN	0.0029014	-1.16343	DOWN	Kdm3b	-2.29	0.00077
10468949	chr2	3424150	3459888	8.60E-10	-1.54473	DOWN	6.30E-10	-1.67969	DOWN	0.00134595	-1.25081	DOWN	Dclre1c	-2.05	0.04331
10439960	chr16	55902263	55934848	1.16E-09	-1.56066	DOWN	1.27E-05	-1.33825	DOWN	0.00229463	-1.24463	DOWN	Cep97	-3.22	0.01066
10376568	chr11	60105013	60199195	1.32E-09	-1.40586	DOWN	0.0235454	-1.1026	DOWN	2.06E-07	-1.43668	DOWN	Rai1	-2.44	0.01987
10421172	chr14	69241846	69503353	1.35E-09	-1.43622	DOWN	2.34E-08	-1.43489	DOWN	0.0663241	1.1058	UP	Slc25a37	-2.26	0.00285
10402554	chr12	107913641	108003414	1.60E-09	-1.30427	DOWN	0.256032	-1.03758	DOWN	7.77E-08	-1.35431	DOWN	Bcl11b	-4.41	0.00338
10507699	chr4	120405409	120530183	1.74E-09	1.50365	DOWN	6.42E-11	-1.77039	DOWN	0.00342906	-1.21532	DOWN	Scmh1	-2.96	0.00177
10549282	chr6	146111464	146501912	2.77E-09	-1.4001	DOWN	0.0242132	-1.10519	DOWN	2.27E-05	-1.3091	DOWN	Itpr2	-2.29	0.00044
10557177	chr7	122289125	122634806	2.98E-09	-1.21269	DOWN	5.18E-07	-1.1807	DOWN	1.91E-05	-1.1701	DOWN	Prkcb	-3.47	0.00045
10380477	chr11	94991212	95006897	3.12E-09	-1.76667	DOWN	0.561478	-1.04234	DOWN	0.0348297	-1.21263	DOWN	Ppp1r9b	-2.84	0.00927
10436734	chr16	87698949	87738408	3.66E-09	-1.40302	DOWN	4.22E-10	-1.56383	DOWN	0.71632	1.01912	DOWN	Bach1	-3.89	0.00007
10362350	chr10	28668360	28882749	3.84E-09	-1.35072	DOWN	2.65E-06	-1.26547	DOWN	2.21E-08	-1.4748	DOWN	Themis	-10.87	0.00571
10350766	chr1	155158742	155203522	3.90E-09	-1.36516	DOWN	1.66E-07	-1.33985	DOWN	0.00074009	-1.20431	DOWN	Stx6	-3.17	0.00109
10522051	chr5	64803523	64830129	3.95E-09	-1.57622	DOWN	0.842898	-1.01149	DOWN	0.0267804	-1.17919	DOWN	Klf3	-2.12	0.00093
10482880	chr2	59899361	60125740	4.37E-09	-1.38275	DOWN	0.682358	1.01707	DOWN	0.00089515	-1.21034	DOWN	Baz2b	-2.12	0.00503
10549615	chr7	4137056	4148173	4.38E-09	-1.53599	DOWN	0.807829	-1.0134	DOWN	5.38E-06	-1.48063	DOWN	Leng8	-2.48	0.00211
10474181	chr2	103566310	103718423	4.51E-09	-1.63634	DOWN	0.0324624	1.15241	UP	1.27E-07	-1.78274	DOWN	Abtb2	-4.72	0.00078
10376534	chr11	59662532	59776222	6.81E-09	-1.27263	DOWN	1.04E-09	-1.36878	DOWN	0.601651	-1.0203	DOWN	Mprip	-3.41	0.00081
10428857	chr15	58941241	59082023	6.89E-09	-1.27116	DOWN	3.22E-07	-1.25028	DOWN	1.04E-06	-1.28677	DOWN	Mtss1	-3.79	0.00918
10551469	chr7	28179483	28187294	9.95E-09	-1.64541	DOWN	0.694335	-1.02652	DOWN	0.00013727	-1.44626	DOWN	Dyrk1b	-3.95	0.00074
10396956	chr12	81860030	82000924	1.08E-08	-1.39488	DOWN	1.18E-05	-1.28183	DOWN	8.17E-05	-1.29594	DOWN	Pcnx	-2.72	0.00389

10378334	chr11	73177083	73182046	1.14E-08	-1.25446	DOWN	1.14E-07	-1.2609	DOWN	0.852568	1.00693	UP	Tax1bp3	-2.34	0.00975
10494428	chr3	96557957	96569602	1.19E-08	-1.63702	DOWN	0.117776	1.11297	UP	2.95E-06	-1.64549	DOWN	Txnip	-3.53	0.00048
10358982	chr1	155127878	155146783	1.21E-08	-1.48732	DOWN	4.33E-11	-1.88358	DOWN	6.02E-08	-1.67544	DOWN	Mr1	-6.85	0.00161
10399265	chrUn	0	0	1.22E-08	-1.27356	DOWN	3.31E-08	-1.30669	DOWN	5.33E-09	-1.43661	DOWN	Ncoa1	-2.08	0.00774
10564290	chr7	63891367	63938915	1.32E-08	-1.55802	DOWN	0.725893	-1.02135	DOWN	2.27E-07	-1.70536	DOWN	Klf13	-3.86	0.00362
10481210	chr2	27262108	27426912	1.63E-08	-1.3419	DOWN	6.08E-07	-1.31804	DOWN	0.208367	-1.06511	DOWN	Vav2	-2.67	0.01447
10389451	chr11	86270986	86357525	2.09E-08	-1.31057	DOWN	0.02531	-1.09357	DOWN	0.0578893	-1.09514	DOWN	Med13	-2.16	0.00931
10373569	chr10	128776742	128777251	2.26E-08	1.29505	UP	6.34E-05	1.19271	UP	4.31E-07	-1.3957	DOWN	Dgka		
10465244	chr19	5800387	5800552	2.28E-08	-1.85682	DOWN	0.585451	1.0486	UP	5.25E-05	-1.68617	DOWN	Malat1	-2.23	0.0064
10591263	chr9	20637786	20644767	2.46E-08	-1.56952	DOWN	2.58E-10	-1.97763	DOWN	7.36E-09	-1.99503	DOWN	Fbxl12	-6.49	0.00003
10464647	chr19	4183411	4191047	2.62E-08	-1.52709	DOWN	0.00020655	1.30014	UP	6.80E-07	-1.63979	DOWN	Tbc1d10c	-2.07	0.00173
10391103	chr11	100370619	100397743	3.23E-08	-1.62529	DOWN	0.236364	-1.0872	DOWN	6.65E-05	-1.50741	DOWN	Jup	-3.14	0.00175
10432006	chr15	97792679	97831671	4.13E-08	-1.35725	DOWN	0.00423616	-1.15002	DOWN	0.00349463	-1.19087	DOWN	Hdac7	-4.22	0.00006
10580139	chr8	84210678	84237055	4.22E-08	-1.6421	DOWN	0.00011129	1.39697	UP	0.0737619	-1.17705	DOWN	Zswim4	-2.6	0.01633
10432190	chr15	98589973	98610088	4.25E-08	-1.51163	DOWN	0.142632	1.09449	UP	4.37E-06	-1.54888	DOWN	Adcy6	-2.3	0.00535
10485388	chr2	101950203	102186360	4.44E-08	-1.73866	DOWN	9.79E-10	-2.23931	DOWN	0.00089453	-1.45168	DOWN	Ldlrad3	-7.19	0.0101
10378240	chr11	72999142	73015200	4.45E-08	-1.63498	DOWN	4.34E-10	-2.11514	DOWN	1.63E-09	-2.3378	DOWN	P2rx1	-4.39	0.01671
10392735	chr11	113726850	113751807	7.41E-08	-1.31171	DOWN	1.45E-11	-1.66495	DOWN	0.0006153	-1.21668	DOWN	Cdc42ep4	-2.03	0.0122
10346330	chr1	55406388	55751463	8.15E-08	-1.43781	DOWN	2.94E-05	-1.32904	DOWN	0.183765	-1.09501	DOWN	Plcl1	-2.88	0.01104
10574789	chr8	105605281	105622219	8.22E-08	-1.35299	DOWN	1.16E-08	-1.48885	DOWN	9.23E-06	-1.37099	DOWN	Fam65a	-3.03	0.00048
10509168	chr4	136172394	136194728	8.64E-08	-1.35437	DOWN	3.25E-10	-1.63005	DOWN	2.72E-06	-1.41425	DOWN	E2f2	-3.83	0.00866
10528548	chr5	24319589	24351604	8.79E-08	-1.45169	DOWN	2.04E-06	-1.42898	DOWN	1.62E-06	-1.55555	DOWN	Kcnh2	-2.94	0.00094
10419814	chr14	54631231	54641318	9.90E-08	-1.50179	DOWN	0.221808	-1.0804	DOWN	8.19E-06	-1.54197	DOWN	Cdh24	-2.84	0.01114
10574259	chr8	94977109	95014196	1.06E-07	1.35179	UP	1.39E-12	-1.91928	DOWN	0.00036654	-1.26427	DOWN	Gpr56	-2.79	0.05411
10432619	chr15	100575318	100599984	1.16E-07	-1.25956	DOWN	0.0266336	-1.08736	DOWN	0.00042579	-1.19477	DOWN	Pou6f1	-3.77	0.0004
10431266	chr15	86139127	86186141	1.18E-07	-1.40645	DOWN	3.16E-10	-1.75201	DOWN	2.17E-06	-1.4969	DOWN	Cerk	-3.13	0.00216
10430519	chr15	79417852	79442002	1.86E-07	-1.2089	DOWN	1.20E-05	-1.18246	DOWN	0.581141	-1.02057	DOWN	Csnk1e	-2.71	0.0032
10462035	chr19	21937283	21938597	1.87E-07	-1.59161	DOWN	1.24E-12	-2.84976	DOWN	0.0170978	-1.25841	DOWN	Ldhb	-6.45	0.0006
10576946	chr8	9970020	9977686	2.03E-07	-1.42859	DOWN	4.04E-10	-1.81944	DOWN	0.0765884	1.1362	UP	Lig4	-6.9	0.0013
10375019	chr11	32000428	32059202	2.69E-07	-1.28766	DOWN	1.25E-12	-1.79047	DOWN	1.67E-10	-1.73621	DOWN	Nsg2	-4.72	0.00043
10462603	chr19	34290666	34327770	3.33E-07	-1.58097	DOWN	2.20E-08	-1.88475	DOWN	6.34E-09	-2.2931	DOWN	Fas	-3.12	0.00392
10435212	chr16	33185071	33243266	5.23E-07	-1.17021	DOWN	3.09E-10	-1.32877	DOWN	0.0003386	-1.146	DOWN	Osbpl11	-2.27	0.00271
10416355	chr14	73142545	73183693	5.79E-07	-1.3014	DOWN	2.57E-11	-1.72755	DOWN	0.00281163	1.2002	UP	Rcbtb2	-3.68	0.00373
10511017	chr4	155409258	155447944	6.09E-07	-1.69306	DOWN	1.10E-09	-2.45362	DOWN	0.00061896	-1.54366	DOWN	2010015L04Rik	-2.21	0.00123
10512480	chr4	43482064	43483722	9.09E-07	-1.28077	DOWN	9.07E-07	-1.33672	DOWN	2.22E-07	-1.47293	DOWN	Sit1	-2.79	0.02613
10410124	chr13	64363214	64370306	9.19E-07	-1.69693	DOWN	1.90E-10	-2.76893	DOWN	0.106605	-1.20796	DOWN	Ctsl	-6.67	0.00427
10555262	chr7	99867351	99917639	1.26E-06	-1.63279	DOWN	1.41E-08	-2.13692	DOWN	0.00163575	-1.46442	DOWN	Xrra1	-5.21	0.0223
10429128	chr15	66780819	66831829	1.39E-06	-1.18971	DOWN	1.29E-12	-1.56035	DOWN	0.229908	-1.04794	DOWN	Sla	-3.23	0.00145
10594879	chr9	71845726	72111803	1.55E-06	-1.12765	DOWN	3.13E-08	-1.19735	DOWN	0.00015224	-1.12811	DOWN	Tcf12	-2.62	0.00044
10540493	chr6	108828652	108859351	2.08E-06	-1.15466	DOWN	2.09E-10	-1.3379	DOWN	0.346843	1.02884	UP	Edem1	-5.95	0.00003
10515613	chr4	118208213	118291397	3.49E-06	-1.19745	DOWN	3.00E-12	-1.6018	DOWN	0.0300536	-1.10207	DOWN	Ptprf	-4.69	0.00031
10498024	chr3	49892514	50443613	3.60E-06	-1.89229	DOWN	1.06E-09	-3.41741	DOWN	0.00099614	-1.75762	DOWN	Slc7a11	-20.83	0.00321
10523297	chr5	93267257	93276225	3.89E-06	-1.2644	DOWN	3.84E-09	-1.52659	DOWN	6.03E-06	-1.38382	DOWN	Ccng2	-4.35	0.00259
10538979	chr6	71322254	71355831	6.29E-06	-1.18635	DOWN	1.20E-08	-1.34835	DOWN	8.49E-14	-1.96999	DOWN	Cd8b1	-40	0.00003
10582123	chr8	119561978	119575183	1.14E-05	-1.30651	DOWN	4.88E-07	-1.47672	DOWN	2.15E-06	-1.53792	DOWN	Hsdl1	-3.56	0.00144
10565852	chr7	99920257	99955531	1.47E-05	-1.19341	DOWN	2.74E-08	-1.36874	DOWN	0.00425778	-1.15671	DOWN	Rnf169	-2.16	0.01722
10453062	chr17	79848392	79896051	1.78E-05	-1.29471	DOWN	5.22E-12	-2.09634	DOWN	0.0350757	-1.16425	DOWN	Atl2	-4.72	0.00025
10462507	chr19	32595849	32667187	1.82E-05	-1.66882	DOWN	8.20E-09	-2.69397	DOWN	0.0650679	-1.29833	DOWN	Papss2	-5.41	0.02056
10378253	chr11	73019043	73042073	1.84E-05	-1.2759	DOWN	4.22E-11	-1.87512	DOWN	2.29E-05	-1.40818	DOWN	Camkk1	-3.12	0.00037
10534889	chr5	137650483	137684693	1.86E-05	-1.2625	DOWN	5.10E-12	-1.9563	DOWN	1.50E-07	-1.58938	DOWN	Agfg2	-5.1	0.00009
10527646	chr5	149368476	149372642	2.20E-05	-1.7568	DOWN	3.53E-08	-2.76406	DOWN	0.00113195	-1.75375	DOWN	BC028471	-9.71	0.00706
10506397	chr4	103114465	103165750	2.96E-05	-1.36088	DOWN	6.62E-08	-1.72967	DOWN	0.541124	1.05355	UP	Mier1	-2.68	0.00465
10403604	chr13	13590409	13777443	3.26E-05	-1.27218	DOWN	8.87E-08	-1.52774	DOWN	0.0313109	-1.16436	DOWN	Lyst	-4.74	0.00083
10494023	chr3	94372786	94398588	3.32E-05	-1.23652	DOWN	8.15E-12	-1.8672	DOWN	3.86E-07	-1.51594	DOWN	Rorc	-22.73	0.00281
10439790	chr16	48731910	48772135	3.88E-05	-1.26266	DOWN	4.46E-06	-1.38002	DOWN	0.0741201	1.12962	UP	Trat1	-2.58	0.01978
10522467	chr5	74195296	74199481	4.52E-05	-1.31858	DOWN	2.53E-10	-2.00785	DOWN	0.591702	-1.0434	DOWN	Rasl11b	-2.51	0.0031
10461057	chr19	7267404	7275225	4.60E-05	1.18302	DOWN	2.23E-11	-1.61932	DOWN	0.00789054	1.14889	UP	Rcor2	-2.51	0.00006
10561712	chr7	29256337	29281912	5.24E-05	-1.14454	DOWN	3.59E-11	-1.46488	DOWN	0.00404619	-1.13173	DOWN	Spint2	-2.96	0.00134
10568691	chr7	134227474	134230705	6.43E-05	-1.6959	DOWN	1.05E-07	-2.64654	DOWN	2.52E-05	-2.2577	DOWN	A130023I24Rik	-5.08	0.02254
10392120	chr11	106263186	106272708	9.03E-05	-1.11605	DOWN	2.37E-11	-1.39396	DOWN	0.00162468	-1.12572	DOWN	Smarcd2	-2.11	0.00183
10445688	chr17	47505131	47599689	0.00011057	-1.14371	DOWN	2.77E-09	-1.3775	DOWN	3.43E-07	-1.3407	DOWN	Ccnd3	-2.6	0.00155
10557575	chr7	127211608	127214288	0.00011893	-1.18372	DOWN	5.70E-09	-1.47519	DOWN	0.0106218	-1.15517	DOWN	Mylpf	-2.75	0.00238
10406598	chr13	92611138	92729806	0.00013046	-1.22855	DOWN	7.06E-09	-1.60411	DOWN	1.92E-08	-1.72224	DOWN	Serinc5	-7.81	0.00009
10579609	chr8	71708636	71721860	0.00015679	1.1762	UP	6.54E-08	-1.3943	DOWN	0.372801	-1.04744	DOWN	Fcho1	-2.18	0.00642
10523670	chr5	103853152	103855316	0.00018411	-1.29786	DOWN	2.02E-07	-1.65789	DOWN	1.56E-10	-1.49999	DOWN	Aff1		
10392970	chr11	115607918	115612929	0.00020517	1.13808	UP	5.95E-14	-1.76544	DOWN	1.60E-06	-1.3097	DOWN	Mif4gd	-2.3	0.00003
10529858	chr5	44175159	44226606	0.00021546	-1.20505	DOWN	5.72E-09	-1.57382	DOWN	0.988539	-1.00088	DOWN	Tapt1	-3.86	0.00122
10597323	chr9	112065258	112234841	0.00051195	-1.21098	DOWN	8.46E-11	-1.89009	DOWN	0.00013989	-1.36241	DOWN	Arpp21	-31.25	0.0012
10547888	chr6	124858444	124863917	0.00052597	-1.19249	DOWN	8.84E-14	-2.29526	DOWN	4.16E-13	-2.55477	DOWN	Gpr162	-9.71	0.00092
10364385	chr10	78574542	78584493	0.00053083	-1.21762	DOWN	5.84E-08	-1.57794	DOWN	0.564025	-1.0415	DOWN	Ilvbl	-2.1	0.00378
10535189	chr5	140008689	140321552	0.00056264	-1.20099	DOWN	4.07E-09	-1.64584	DOWN	0.00040301	-1.31072	DOWN	Mad1l1	-2.11	0.01731
10444431	chr17	34629686	34632260	0.00076296	-1.15801	DOWN	7.93E-12	-1.77654	DOWN	3.28E-05	-1.32187	DOWN	Prrt1	-3.16	0.0082
10548735	chr6	134715468	134792628	0.00134872	-1.12292	DOWN	7.08E-12	-1.62586	DOWN	4.83E-05	1.25533	UP	Dusp16	-2.16	0.0075
10572815	chrUn	0	0	0.00220096	-1.16608	DOWN	1.34E-09	-1.67684	DOWN	1.00E-05	-1.43425	DOWN	Tmem38a	-3.47	0.00281
10406551	chr13	91461089	91703429	0.00228767	-1.14188	DOWN	2.77E-11	-1.736	DOWN	1.06E-07	-1.52878	DOWN	Ssbp2	-6.8	0.00014
10513869	chr4	70428853	70534928	0.00240346	1.14771	UP	6.22E-11	-1.73744	DOWN	0.662651	1.02572	UP	Megf9	-2.01	0.03396
10522186	chr5	65967448	65977247	0.00273656	-1.25279	DOWN	3.77E-11	-2.56659	DOWN	0.00010799	-1.56681	DOWN	Chrna9	-3.05	0.00319
10564451	chr7	67232084	67372858	0.00309264	-1.1341	DOWN	2.21E-08	-1.45734	DOWN	0.00037602	-1.25477	DOWN	Mef2a	-2.27	0.0014
10553788	chr7	58658332	58829420	0.00469444	-1.14916	DOWN	9.42E-11	-1.81199	DOWN	1.01E-06	-1.5256	DOWN	Atp10a	-2.48	0.00525
10380275	chr11	89064729	89069098	0.00600891	-1.1842	DOWN	6.01E-09	-1.81515	DOWN	0.00230185	-1.31502	DOWN	A930013B10Rik	-12.82	0.0025
10400795	chr12	69965012	69987002	0.00863452	-1.14229	DOWN	2.85E-10	-1.79525	DOWN	0.556115	1.0402	UP	Sav1	-2.02	0.00073
10366038	chr10	99108138	99113247	0.0093393	-1.18748	DOWN	1.26E-05	-1.48551	DOWN	0.0130684	-1.26205	DOWN	Galnt4	-2.44	0.00796
10426479	chr15	95790843	95974774	0.011695	-1.10543	DOWN	6.22E-08	-1.40619	DOWN	0.608575	-1.02743	DOWN	Ano6	-3.32	0.00004
10543572	chr6	29200437	29216350	0.0117596	-1.10853	DOWN	4.27E-06	-1.30519	DOWN	0.839996	1.01101	UP	Impdh1	-3.07	0.00993
10538993	chr6	71373427	71377609	0.0152793	-1.21256	DOWN	6.22E-15	-4.93256	DOWN	4.29E-06	-1.88502	DOWN	Cd8a	-71.43	0.00008
10429520	chr15	74762056	74763567	0.0191509	1.19204	UP	4.97E-12	-2.96852	DOWN	0.270734	-1.11871	DOWN	Ly6d	-12.66	0.00103
10361897	chr10	19591958	19610229	0.0205253	-1.08389	DOWN	1.42E-09	-1.45495	DOWN	0.00023495	-1.22414	DOWN	Ifngr1	-4.13	0.00055
10355214	chr1	65158628	65179114	0.0246739	-1.22231	DOWN	1.24E-06	-1.90365	DOWN	0.590794	1.06705	UP	Idh1	-2.98	0.002
10450226	chr17	34616662	34627346	0.0254539	-1.09015	DOWN	2.52E-08	-1.42666	DOWN	0.128764	-1.08459	DOWN	Ppt2	-2.69	0.00254
10494821	chr3	102734529	102772292	0.0278018	1.20844	UP	1.68E-10	-2.85227	DOWN	0.544548	-1.07323	DOWN	Tspan2	-6.41	0.00969
10536390	chr6	8259444	8597548	0.0371581	-1.10554	DOWN	7.26E-09	-1.61557	DOWN	8.42E-10	-1.60801	DOWN	Glcci1	-2.03	0.00002
10405216	chr13	52583501	52648792	0.0906595	-1.06151	DOWN	1.14E-10	-1.56713	DOWN	0.0175766	1.13173	UP	Syk	-2.1	0.00334
10418729	chr14	31524296	31577317	0.116634	-1.08724	DOWN	1.02E-10	-1.98565	DOWN	0.225463	-1.09555	DOWN	Colq	-8.85	0.00339
10362581	chr10	39134023	39151058	0.148642	-1.09894	DOWN	7.02E-11	-2.36522	DOWN	0.0172171	-1.26116	DOWN	Tube1	-9.01	0.0009
10429515	chr15	74747856	74752988	0.161347	-1.0982	DOWN	2.49E-05	-1.49564	DOWN	0.284439	-1.10663	DOWN	Lynx1	-2.46	0.01377
10523468	chr5	96997689	97091048	0.186082	1.0563	UP	5.71E-08	-1.45821	DOWN	0.181692	1.0823	UP	Bmp2k	-2.08	0.04264
10408613	chr13	34127008	34130595	0.215622	-1.11017	DOWN	1.48E-07	-2.06856	DOWN	0.723019	1.04808	UP	Tubb2b	-3.27	0.00483
10458960	chr18	56525736	56572872	0.226352	-1.04276	DOWN	3.98E-09	-1.44656	DOWN	0.0130921	1.13857	UP	Aldh7a1	-2.79	0.00088

**10474105**	**chr2**	**101624748**	**101632528**	**0.261693**	**-1.14582**	**DOWN**	**2.64E-08**	**-3.19921**	**DOWN**	**4.68E-08**	**-3.93405**	**DOWN**	**Rag2**		**-47.62**	**0.00395**
**10534596**	**chr5**	**136248135**	**136567431**	**0.274731**	**-1.02497**	**DOWN**	**8.98E-09**	**-1.25952**	**DOWN**	**0.566765**	**1.01847**	**UP**	**Cux1**		**-2.13**	**0.00159**
**10499138**	**chr3**	**86786153**	**86920852**	**0.420191**	**-1.02865**	**DOWN**	**2.11E-09**	**-1.47836**	**DOWN**	**0.50914**	**1.03352**	**UP**	**Dclk2**		**-2.02**	**0.01176**
**10537567**	**chr6**	**41047340**	**41548352**	**0.459388**	**1.0116**	**UP**	**7.09E-08**	**-1.15265**	**DOWN**	**0.00818162**	**-1.06566**	**DOWN**	**Tcrb-J**		**-4**	**0.00063**
**10362896**	**chr10**	**43579169**	**43584267**	**0.497684**	**-1.03332**	**DOWN**	**3.28E-08**	**-1.58689**	**DOWN**	**4.74E-07**	**1.61151**	**UP**	**Cd24a**		**-11.49**	**0.00002**
**10500847**	**chr3**	**104014969**	**104220027**	**0.634268**	**1.03083**	**UP**	**3.64E-08**	**-1.83633**	**DOWN**	**0.0235347**	**-1.24466**	**DOWN**	**Magi3**		**-3.36**	**0.00014**
**10445412**	**chr17**	**45555527**	**45563656**	**0.642886**	**1.03089**	**UP**	**3.39E-08**	**-1.87269**	**DOWN**	**0.00054151**	**1.45337**	**UP**	**Nfkbie**		**-2.38**	**0.00045**
**10604230**	**chrX**	**38685589**	**38686170**	**0.675428**	**-1.01792**	**DOWN**	**8.71E-07**	**-1.39283**	**DOWN**	**0.109193**	**1.10497**	**UP**	**Ap3s1**		**-2.44**	**0.0035**
**10532124**	**chr5**	**107716659**	**107725805**	**0.795441**	**-1.00795**	**DOWN**	**2.72E-13**	**-1.72696**	**DOWN**	**0.0118695**	**-1.1255**	**DOWN**	**Gfi1**		**-3.11**	**0.00052**
**10400141**	**chr12**	**44619186**	**44620361**	**0.893616**	**1.00661**	**UP**	**1.25E-07**	**-1.54658**	**DOWN**	**0.100269**	**1.11748**	**UP**	**Zbed4**		**-2.98**	**0.00484**
**10474048**	**chr2**	**92915101**	**92956051**	**0.950362**	**1.00301**	**UP**	**3.36E-10**	**-1.82204**	**DOWN**	**0.489623**	**-1.04909**	**DOWN**	**Syt13**		**-2.55**	**0.00337**
**10523012**	**chr5**	**88765027**	**88783277**	**0.984309**	**1.00071**	**UP**	**3.41E-11**	**-1.65805**	**DOWN**	**0.841056**	**-1.01039**	**DOWN**	**Dck**		**-3.95**	**0.00149**
**10601091**	**chrX**	**101254528**	**101259686**	**0.991416**	**1.00045**	**UP**	**1.74E-08**	**-1.51417**	**DOWN**	**0.71148**	**-1.02227**	**DOWN**	**Foxo4**		**-2.27**	**0.00356**

Supplementary Table 2: Genes from our dataset in common with Cohen's upregulated genes by Stage 1 (Cohen et al, 2012)

				Regulation in T1			Regulation in T2			Regulation in T3				Cohen et al 2012: Genes	
probeset_id	seqname	start	stop	p-value(DPT vs. DPNKT)	FC to DPNKT	Direction in DPNKT	p-value(DPNKT vs. CD4NKT)	FC to CD4NKT	Direction in CD4NKT	p-value(CD4NKT vs. DNNKT)	FC to DNNKT	Direction in DNNKT	Gene Symbol	FC (Stage vs DP)	FC(CD4+8int)/DP)
10552406	chr7	43437073	43438249	2.01E-05	1.43283	UP	2.57E-23	35.7082	UP	4.29E-05	-1.62253	DOWN	Nkg7	3	1.56
10530145	chr5	64924686	64932699	0.00320613	1.18557	UP	3.78E-23	15.0944	UP	0.00013844	-1.40283	DOWN	Tlr1	4.85	1.54
10531724	chr5	100553726	100572225	5.22E-10	1.9229	UP	4.52E-20	10.7596	UP	1.40E-07	1.96865	UP	Plac8	14.48	1.46
10574246	chr8	94923694	94943280	0.0516835	-1.14409	DOWN	1.19E-20	14.1103	UP	1.85E-11	-3.21267	DOWN	Gpr114	4.47	1.24
10404359	chr13	30136490	30246698	1.89E-15	2.32137	UP	3.70E-12	1.97518	UP	0.00539344	1.20785	UP	Mboat1	4.45	1.97
10593225	chr9	48654297	48835945	0.110438	-1.13292	DOWN	2.85E-19	13.7356	UP	0.60439	-1.05798	DOWN	Zbtb16	37.64	1.82
10555510	chr7	101421709	101512819	0.269162	-1.07466	DOWN	4.25E-19	8.82058	UP	0.0358948	-1.2249	DOWN	Pde2a	2.32	1.07
10378286	chr11	73090583	73147441	0.698008	-1.02268	DOWN	7.90E-19	6.68302	UP	5.41E-13	-3.37665	DOWN	Itgae	17.37	1.46
10487208	chr2	126322810	126491553	0.00094688	1.30845	UP	1.66E-17	7.65041	UP	9.04E-05	1.61786	UP	Atp8b4	17.12	1.11
10525419	chr5	122643911	122691432	0.168082	1.09078	UP	4.76E-18	6.46401	UP	0.161268	-1.13459	DOWN	P2rx7	3.17	1.56
10552500	chr7	43797577	43803822	0.0163742	-1.13908	DOWN	4.89E-19	5.50639	UP	3.10E-05	-1.45306	DOWN	Klk8	3.83	1.89
10368289	chr10	24641411	24712102	0.788113	1.01401	UP	5.97E-18	4.70625	UP	2.30E-10	2.22388	UP	Enpp1	3.37	1.2
10363231	chr10	57794544	57811830	0.170391	1.1029	UP	2.19E-17	7.16536	UP	0.715502	-1.03713	DOWN	Smpdl3a	21.13	1.64
10590623	chr9	123806475	123811760	0.501817	-1.05798	DOWN	8.69E-18	11.6643	UP	0.0424564	-1.2892	DOWN	Cxcr6	12.37	1.66
10402136	chr12	100876685	100887881	0.626842	-1.04422	DOWN	1.25E-17	13.0411	UP	0.00013981	-1.78006	DOWN	Gpr68	3.91	0.94
10427235	chr15	102459175	102462784	0.47529	1.03321	UP	8.45E-17	3.32875	UP	0.151473	1.10021	UP	Prr13	3.25	0.98
10600901	chrX	98149750	98317147	0.0516312	-1.18509	DOWN	1.54E-17	10.9254	UP	5.13E-08	-2.58882	DOWN	Ar	4.02	1.19
10371356	chr10	83600033	83648664	0.447403	-1.04245	DOWN	4.30E-17	4.4092	UP	1.38E-07	-1.79218	DOWN	Appl2	5.3	1.84
10454015	chr18	12643783	12737051	0.918136	-1.00608	DOWN	7.24E-17	4.80636	UP	0.813927	1.02004	UP	Ttc39c	4.58	1.04
10568328	chr7	127893063	127895617	1.16E-13	1.95779	UP	1.09E-08	1.54055	UP	0.00017922	1.30735	UP	Vkorc1	2.15	1.17
10558631	chr7	140247301	140297641	0.00123428	1.2094	UP	2.81E-15	3.19312	UP	9.66E-05	1.41664	UP	5830411N06Rik	2.16	1.08
10590631	chr9	124102183	124108550	0.943174	1.00651	UP	1.77E-16	10.1955	UP	0.497953	1.0926	UP	Ccr2	8.13	1.01
10586781	chr9	70207350	70400081	0.359099	-1.05917	DOWN	1.62E-16	4.91031	UP	0.00220886	1.3549	UP	Myo1e	11.48	1.56
10471457	chr2	32587490	32600492	1.25E-10	1.42182	UP	6.70E-11	1.53184	UP	2.12E-08	1.45984	UP	St6galnac4	3	1.85
10590909	chr9	14354005	14381242	0.00089692	1.36693	UP	2.42E-14	5.26584	UP	0.00338128	1.46573	UP	Endod1	9.72	1.44
10466127	chr19	11047980	11048398	3.59E-07	1.74334	UP	1.12E-12	3.71291	UP	0.942225	1.00817	UP	AW112010	5.37	1.35
10489891	chr2	167298444	167349183	9.89E-10	1.70318	UP	6.98E-11	2.05377	UP	0.54902	-1.04687	DOWN	B4galt5	2.62	1.08
10447773	chr17	12419971	12507704	2.07E-07	1.38745	UP	2.77E-12	2.0404	UP	6.55E-15	3.2239	UP	Slc22a3	4.79	1.16
10458906	chr18	53406341	53418022	3.38E-05	1.24771	UP	2.34E-13	2.17952	UP	0.00456705	1.21242	UP	Ppic	2.19	1.09
10389300	chr11	84820726	84828986	6.01E-05	1.2704	UP	2.01E-13	2.43425	UP	1.04E-07	1.70637	UP	Dhrs11	2.86	0.97
10553598	chr7	55842027	55930698	0.268376	1.04037	UP	5.48E-15	2.14607	UP	0.236141	1.06252	UP	Cyfip1	3.43	1.64
10480238	chr2	13654929	13793520	0.912744	-1.00917	DOWN	1.81E-15	6.70571	UP	1.80E-08	-2.7451	DOWN	St8sia6	8.38	1.74
10578123	chr8	33782644	33929841	0.886911	1.00978	UP	2.79E-15	4.62681	UP	4.80E-06	1.7875	UP	Rbpms	11.46	1.12
10372781	chr10	120141654	120201537	0.0165272	-1.25696	DOWN	2.91E-16	9.22756	UP	0.798874	1.03301	UP	Irak3	2.07	0.96
10476945	chr2	150570415	150578942	0.0926895	-1.1495	DOWN	6.40E-16	6.85609	UP	0.330216	-1.11938	DOWN	Cst7	6.08	1.12
-10496438	chr3	138277645	138290698	0.166261	-1.1245	DOWN	1.25E-15	6.87501	UP	3.02E-05	-1.84508	DOWN	Adh1	8.54	0.99
10492426	chr3	65393337	65395184	0.00289273	1.23666	UP	1.73E-13	3.23596	UP	0.715785	-1.03397	DOWN	Acsf2	2.18	1.25
10390032	chr11	94557102	94601871	0.00363724	1.23857	UP	1.66E-13	3.38779	UP	0.655476	-1.04341	DOWN	Acsf2		
10512774	chr4	46537559	46566440	0.849825	1.01968	UP	1.24E-14	8.51871	UP	0.157555	1.23695	UP	Coro2a	3	1.49
10568553	chr7	132235780	132317155	2.50E-05	1.37964	UP	6.53E-12	2.55005	UP	0.00357046	1.32648	UP	Chst15	2.02	1.98
10447056	chr17	79051906	79090243	0.55186	1.03364	UP	3.62E-14	2.98938	UP	0.00053714	1.37261	UP	Qpct	3.31	1.41
10481092	chr2	26593090	26604291	2.48E-06	1.42773	UP	5.73E-11	2.18429	UP	9.34E-07	1.72206	UP	Agpat2	4.37	1.39
10355998	chr1	80198706	80213944	0.0659158	-1.14975	DOWN	5.88E-15	4.82886	UP	0.952073	1.00628	UP	Fam124b	2.17	1.31
10381272	chr11	101176117	101190720	0.0259311	-1.21256	DOWN	4.02E-15	6.01856	UP	0.124305	1.20232	UP	Cntnap1	5.44	0.86
10460385	chr19	4214392	4222615	0.261483	-1.07075	DOWN	1.95E-14	3.39513	UP	6.44E-06	-1.64617	DOWN	Clcf1	2.59	1.7
10437687	chr16	10958859	10993307	3.44E-07	1.37628	UP	6.33E-10	1.71755	UP	0.0620253	1.13326	UP	Litaf	2.26	1.09
10425053	chr15	78244834	78262580	0.784179	-1.01379	DOWN	6.34E-14	2.61543	UP	0.00046704	-1.33618	DOWN	Ncf4	3.89	1.08
10352178	chr1	179668231	179687186	0.014988	1.20151	UP	1.72E-12	3.15564	UP	4.19E-10	2.86605	UP	Sccpdh	4.59	1.01
10368647	chr10	34151393	34207551	0.690452	-1.02369	DOWN	6.26E-14	3.09487	UP	1.37E-06	-1.72169	DOWN	Dse	2.39	1.41
10497441	chr3	20122084	20155096	0.0551224	1.10441	UP	1.05E-12	2.2938	UP	7.10E-06	1.50549	UP	Gyg	2.18	1.11
10500406	chr3	96800155	96814493	0.220668	-1.07572	DOWN	5.97E-14	3.09512	UP	4.46E-05	1.52099	UP	Cd160	2.36	1.07
10454286	chr18	23803984	23893857	8.52E-07	1.56668	UP	1.87E-09	2.13797	UP	4.39E-05	1.61836	UP	Mapre2	6.8	1.45
10456745	chr18	75367365	75395934	0.550153	-1.05402	DOWN	1.95E-13	4.93439	UP	0.00408644	-1.48729	DOWN	Smad7	3.05	1.72
10367945	chr10	13213395	13474396	0.258909	1.08376	UP	1.88E-12	3.13158	UP	0.00423443	-1.37207	DOWN	Phactr2	5.19	1.99
10470446	chr2	27677201	27763319	0.00467951	1.18633	UP	2.49E-11	2.18762	UP	0.00613022	-1.26513	DOWN	Rxra	2.48	1.05
10376324	chr11	58187759	58189012	0.10319	1.15962	UP	3.98E-12	3.97277	UP	0.00206449	-1.54432	DOWN	Gm12250	3.55	1.77
10424404	chr15	62037986	62242562	3.29E-06	1.4205	UP	2.36E-09	1.90195	UP	0.00062066	1.38293	UP	Pvt1	6.33	1.91
10581992	chr8	115693159	115706894	0.818335	1.01414	UP	1.61E-12	2.72109	UP	3.29E-07	1.86012	UP	Maf	3.42	1.95
10596148	chr9	103208874	103230286	0.39616	1.04662	UP	3.73E-12	2.30842	UP	2.21E-10	2.28115	UP	Trf	2.56	1.28
10504692	chr4	46060966	46114804	0.465722	-1.05053	DOWN	9.01E-13	3.10527	UP	0.00011751	1.55744	UP	Tmod1	2.49	1.02
10397763	chr12	100779106	100872621	0.00033313	1.23208	UP	1.02E-09	1.7897	UP	0.474277	1.0525	UP	9030617O03Rik	3.67	1.68
10379176	chr11	78343505	78349156	0.208163	1.07884	UP	2.58E-11	2.32142	UP	1.78E-05	1.57733	UP	Unc119	2.77	0.92
10388958	chr11	79513385	79530609	0.169745	1.0744	UP	3.74E-11	2.04129	UP	3.70E-05	1.45027	UP	Evi2a	2.28	1.05
10558614	chr7	140218267	140231145	0.861095	1.01351	UP	1.25E-11	3.10363	UP	0.0460403	1.25744	UP	Cd163l1	2.22	1.22
10596637	chr9	107254927	107289877	0.142393	-1.11335	DOWN	1.72E-12	3.2094	UP	0.204408	-1.14098	DOWN	Mapkapk3	4.46	1.97
10519983	chr5	21372673	21378373	0.0494495	-1.13712	DOWN	1.38E-12	2.80748	UP	0.00077188	1.26432	UP	Fhl2	2.77	1.12
10391373	chr11	101172998	101175443	0.136094	-1.14266	DOWN	3.00E-12	3.99346	UP	0.00137082	1.5699	UP	Ccr10	2.34	1.08
10464586	chr19	4040294	4042221	4.31E-08	1.45793	UP	2.77E-05	1.33058	UP	0.00174022	1.26545	UP	Gstp2	2.14	1.18
10372028	chr10	94793186	94944578	2.70E-05	1.39067	UP	1.50E-07	1.73988	UP	0.180538	-1.13146	DOWN	Plxnc1	3.75	1.15
10533095	chr5	118064999	118155385	0.0129065	1.16164	UP	2.53E-09	1.86504	UP	0.509146	-1.05453	DOWN	Fbxw8	2.93	1.38
10357249	chr1	120226423	120273188	0.372397	-1.06704	DOWN	1.54E-11	2.86627	UP	0.0473563	1.23848	UP	Steap3	2.3	0.8
10395039	chr12	26469204	26479837	0.599631	1.03977	UP	1.21E-10	2.64303	UP	0.0101777	-1.34193	DOWN	Cmpk2	2.8	1.03
10357833	chr1	133706646	133753281	0.829132	1.01349	UP	8.39E-11	2.29447	UP	0.874709	-1.01409	DOWN	Atp2b4	2.92	1.26
10509777	chr4	139531689	139617112	0.0499701	-1.0953	DOWN	4.15E-12	2.00073	UP	0.0728031	-1.12543	DOWN	Iffo2	2.71	1.63
10440491	chr16	84954443	85173703	0.246335	1.04891	UP	3.54E-10	1.65286	UP	3.74E-08	-1.60155	DOWN	App	3.04	0.93
10373902	chr11	4218251	4222409	0.893101	-1.00807	DOWN	5.63E-11	2.26464	UP	0.762167	-1.02624	DOWN	Gatsl3	2.04	1
10593723	chr9	54604890	54661885	0.479349	-1.02739	DOWN	3.77E-11	1.69865	UP	1.04E-06	-1.43069	DOWN	Acsbg1	2.52	1.04
10375402	chr11	46056052	46147343	0.0225902	-1.14307	DOWN	4.63E-12	2.35587	UP	2.66E-09	-2.10793	DOWN	Adam19	8.53	1.54
10361926	chr10	19934472	20141723	3.27E-06	1.51395	UP	5.11E-06	1.60179	UP	0.976651	-1.00285	DOWN	Map3k5	3.91	1.51
10495405	chr3	109123149	109168457	0.371286	1.07096	UP	5.77E-10	2.50162	UP	0.115764	1.19211	UP	Slc25a24	5.54	1.38
10543785	chr6	31165511	31218433	0.573194	-1.06831	DOWN	7.69E-11	4.80935	UP	0.021102	-1.50686	DOWN	AB041803	5.7	1.2
10538901	chr6	68244798	68245115	0.84492	1.01418	UP	2.39E-10	2.48618	UP	0.392971	1.09262	UP	BC005685	3.74	1.28
10514128	chr4	83221951	83225127	0.965657	1.00265	UP	1.94E-10	2.19461	UP	0.606049	1.04644	UP	Ttc39b	7.14	1.85
10571344	chr8	36094861	36147785	3.88E-12	-1.72883	DOWN	0.627741	1.02362	UP	0.00429907	-1.20217	DOWN	D8Ertd82e	2.49	1.97
10429607	chr15	75657033	75678790	0.00249252	1.13994	UP	5.78E-08	1.43397	UP	3.35E-08	1.57428	UP	Top1mt	2.36	1.6
10406672	chr13	93940990	93943016	0.428217	-1.07346	DOWN	9.21E-11	3.26263	UP	0.486945	-1.09278	DOWN	Arsb	3.83	0.83
10361139	chrUn	0	0	3.37E-05	1.35543	UP	2.06E-06	1.55008	UP	0.0399465	-1.20083	DOWN	Traf5	3.21	1.24
10409767	chr13	59634996	59675784	0.958519	-1.00467	DOWN	4.21E-10	3.00058	UP	0.15476	-1.20478	DOWN	Golm1	3.37	1.51
10461369	chr19	8989284	9076919	0.00027152	-1.19835	DOWN	1.61E-12	2.00001	UP	0.00017268	-1.30946	DOWN	Ahnak	4.11	1.43
10506188	chr4	99929480	99987294	0.0115986	1.1699	UP	4.99E-08	1.71608	UP	0.00020729	1.43527	UP	Pgm2	3.12	1.25
10455901	chr18	58556240	58612869	0.714525	1.02474	UP	9.90E-10	2.18561	UP	0.26888	1.11283	UP	Slc27a6	2.72	1.52
10514133	chr4	83226842	83324189	0.6547	-1.02553	DOWN	3.71E-10	2.00402	UP	0.196277	1.11167	UP	Ttc39b	7.14	1.85
10496338	chr3	136670770	136937386	0.0557105	-1.11716	DOWN	4.01E-11	2.16267	UP	0.397462	1.06997	UP	Ppp3ca	4.98	1.83
10548030	chr6	125460266	125494760	0.0482295	1.20315	UP	3.12E-08	2.36692	UP	0.500417	-1.09042	DOWN	Cd9	9.5	1.9

10440393	chr16	75858793	75953026	3.33E-07	-1.29128	DOWN	1.77E-13	1.93075	UP	5.67E-07	1.42327	UP	Samsn1	2.82	1.96
10483679	chr2	73343460	73386398	0.433962	-1.05422	DOWN	7.22E-10	2.2233	UP	0.0240132	-1.25794	DOWN	Gpr155	3.02	1.1
10531195	chr5	89710657	89710740	2.83E-05	-1.54944	DOWN	3.94E-05	1.64872	UP	4.66E-06	2.04668	UP	Adamts3	2.08	1.29
10587683	chr9	88956900	88962416	0.314729	-1.0714	DOWN	7.33E-10	2.24303	UP	6.35E-05	-1.6025	DOWN	Bcl2a1d	4.88	1.39
10476042	chr2	130012349	130048561	0.822585	1.01435	UP	5.65E-09	1.9649	UP	1.23E-06	1.8076	UP	Tgm3	4.48	1.35
10442219	chr17	21535539	21562616	0.0218473	1.34383	UP	2.92E-07	2.76696	UP	0.0281837	1.49485	UP	Zfp52	3.05	1.54
10523595	chr5	103425179	103598352	0.0972115	-1.09416	DOWN	3.51E-10	1.91828	UP	2.97E-08	1.8563	UP	Ptpn13	2.94	1.29
10493820	chr3	90612894	90614415	0.208783	-1.0889	DOWN	7.52E-10	2.20656	UP	0.00033023	-1.49048	DOWN	S100a6	8.22	0.95
10363415	chr10	60106257	60135196	0.0427213	-1.17385	DOWN	2.25E-10	2.60369	UP	0.0243582	-1.29359	DOWN	Spock2	2.17	1.42
10589884	chr9	114330135	114330578	0.751275	1.02621	UP	1.10E-08	2.3043	UP	0.174661	-1.17533	DOWN	Bcl2a1c	5.4	1.4
10490826	chr3	9251293	9281823	0.278152	-1.0614	DOWN	1.69E-09	1.85299	UP	5.00E-07	1.70942	UP	Zbtb10	3.95	1.28
10587503	chr9	83548330	83600292	0.207838	-1.09717	DOWN	1.57E-09	2.28406	UP	0.0027167	-1.41173	DOWN	Sh3bgrl2	2.66	1.27
10550509	chr7	18884680	18890441	0.478863	-1.04138	DOWN	4.31E-09	1.84946	UP	0.3683	1.07668	UP	Pglyrp1	4.1	1.52
10481962	chr2	34983331	35061396	0.102294	1.06916	UP	3.44E-07	1.39085	UP	0.0805104	1.10815	UP	Hc	2.71	1.03
10573319	chr8	84125989	84132567	0.768183	1.01613	UP	3.68E-08	1.67752	UP	0.919158	1.0079	UP	Podnl1	3.42	0.82
10388042	chr11	71031941	71033513	0.515675	1.04412	UP	1.13E-07	1.80294	UP	0.0425201	-1.22271	DOWN	6330403K07Rik	3.03	1.03
10468639	chr19	56529161	56548222	0.0302897	1.13301	UP	2.57E-06	1.48714	UP	0.00056499	1.36382	UP	Dclre1a	2.26	1.41
10463070	chr19	40659794	40741602	0.131961	1.07237	UP	7.10E-07	1.43104	UP	0.0731047	1.12761	UP	Entpd1	2.54	1.4
10576391	chr8	123806025	123835287	0.238563	-1.05209	DOWN	1.27E-08	1.53793	UP	0.02323	1.15732	UP	Rab4a	3.01	1.31
10422028	chrUn	0	0	0.643209	1.02687	UP	2.57E-07	1.62215	UP	0.791728	1.0218	UP	Tbc1d4	2.49	1.6
10492971	chr3	87376448	87402934	4.64E-06	-1.32849	DOWN	1.11E-11	2.03217	UP	3.61E-07	1.61935	UP	Fcrl1	2.63	1.33
10461553	chr19	10740947	10786043	0.0143234	-1.18676	DOWN	1.69E-09	2.09847	UP	0.0700773	1.19144	UP	A430093F15Rik	2.21	1.99
10385837	chr11	53631324	53634702	0.077915	-1.11354	DOWN	1.13E-08	1.82479	UP	0.838598	1.01728	UP	Il13	5.99	1.01
10360806	chr1	182467259	182517524	0.389306	1.04071	UP	1.62E-06	1.41222	UP	0.49774	-1.04578	DOWN	Capn2	3.14	1.71
10575034	chr8	106510891	106556908	0.0337871	-1.13572	DOWN	8.47E-09	1.80575	UP	0.00020763	1.42814	UP	Cdh3	2.27	1.16
10480714	chr2	25360356	25365626	0.69541	1.01441	UP	1.53E-06	1.31662	UP	0.00328627	1.18523	UP	Uap1l1	2.65	1.27
10584334	chr9	37555698	37648318	0.0354598	1.23573	UP	7.84E-05	1.7086	UP	0.0148734	1.42854	UP	Siae	3.02	1.37
10345698	chr1	39368149	39371907	1.09E-05	1.16216	UP	0.0844144	1.0579	UP	0.168501	1.05552	UP	Npas2	2.77	0.61
10417095	chr14	121035574	121285793	0.131649	-1.08312	DOWN	6.51E-08	1.6088	UP	0.906971	-1.00865	DOWN	Farp1	3.17	0.93
10389879	chr11	94237598	94242549	0.361879	-1.05478	DOWN	2.07E-07	1.64454	UP	0.00027016	-1.42511	DOWN	Wfikkn2	3.2	1.32
10432972	chr15	102234940	102257483	0.354115	-1.05686	DOWN	2.63E-07	1.64851	UP	1.95E-05	-1.57125	DOWN	Rarg	3.37	1.44
10551464	chr7	28169710	28179269	3.87E-07	1.4575	UP	0.704466	1.02413	UP	0.0421858	1.17881	UP	Fbln1	2.18	1.13
10440099	chr16	58468125	58524246	2.59E-10	-1.91377	DOWN	2.14E-13	2.98734	UP	0.00024377	-1.45202	DOWN	St3gal6	2.05	1.68
10367076	chr10	128015172	128030030	3.62E-13	2.77581	UP	6.45E-07	-1.71841	DOWN	0.00023784	1.52141	UP	Prim1	2.61	0.47
10594825	chr9	71110659	71163289	0.0346995	-1.19059	DOWN	6.51E-08	2.05999	UP	7.39E-05	-1.71296		Aqp9	4.28	1.76
10548307	chr6	128778485	128788641	0.551355	-1.04012	DOWN	1.81E-06	1.63274	UP	0.0117184	1.29096	UP	Klrb1c	4.23	0.79
10406270	chr13	75839910	75850151	0.0770661	-1.12512	DOWN	2.19E-07	1.7334	UP	0.121015	1.15774	UP	Glrx	4.8	1.96
10506680	chr4	107367784	107414341	2.86E-12	1.8345	UP	1.59E-06	-1.39943	DOWN	0.00010431	1.34703	UP	Tmem48	2.3	1.31
10477325	chr2	153649454	153729907	0.830313	1.0097	UP	6.51E-05	1.29216	UP	2.78E-07	1.58756	UP	Dnmt3b	2.08	1.35
10486396	chr2	120089487	120154575	2.30E-12	1.80313	UP	8.54E-07	-1.40023	DOWN	1.31E-06	1.48974	UP	Ehd4	2.14	0.76
10389395	chr11	86058136	86201193	7.89E-12	2.41106	UP	3.27E-06	-1.6296	DOWN	0.0941703	1.18371	UP	Brip1	2.35	0.8
10356299	chr1	85939537	85961055	0.0235918	-1.12592	DOWN	8.70E-07	1.47126	UP	3.87E-06	-1.52947	DOWN	Gpr55	2.33	1.14
10592655	chr9	42963843	43107239	2.25E-06	-1.33806	DOWN	1.36E-09	1.70839	UP	0.680082	1.02783	UP	Arhgef12	2.28	1.05
10360070	chr1	171229572	171234349	0.0407353	-1.20702	DOWN	2.02E-06	1.91088	UP	0.00054894	1.64776	UP	Fcer1g	2.53	1.21
10396671	chr12	76533582	76579042	2.60E-13	1.75055	UP	1.12E-08	-1.45376	DOWN	0.703541	1.02012	UP	Plekhg3	2.69	0.98
10452815	chr17	73883908	73950182	6.80E-06	-1.26274	DOWN	4.13E-09	1.54663	UP	0.00023067	-1.28404	DOWN	Xdh	2.41	0.81
10407435	chr13	4132615	4150631	0.118574	-1.09658	DOWN	1.40E-05	1.44707	UP	0.501014	1.05706	UP	Akr1c18	10.63	1.23
10595404	chr9	85324457	85326768	3.09E-07	-1.49953	DOWN	0.0531443	1.1439	UP	0.0131912	-1.24127	DOWN	Fam46a	2.85	1.61
10564667	chr7	78192559	78577975	5.99E-10	-1.56095	DOWN	1.19E-05	1.32644	UP	1.25E-09	-1.84287	DOWN	Ntrk3	2.85	0.76
10567412	chr7	119772327	119793960	2.77E-09	2.03163	UP	4.54E-05	-1.55348	DOWN	0.294439	1.12065	UP	Eri2	2.06	1.07
10437970	chr16	15889224	16146833	8.04E-10	1.61072	UP	3.74E-06	-1.39681	DOWN	0.0596804	1.14141	UP	2310008H04Rik	2.06	1.56
10440284	chr16	64765732	64770939	0.0790562	1.15922	UP	0.881519	-1.01429	DOWN	0.310372	-1.0813	DOWN	4930452B06Rik	2.02	0.78
